# The small G-protein RalA promotes progression and metastasis of triple-negative breast cancer

**DOI:** 10.1186/s13058-021-01438-3

**Published:** 2021-06-12

**Authors:** Katie A. Thies, Matthew W. Cole, Rachel E. Schafer, Jonathan M. Spehar, Dillon S. Richardson, Sarah A. Steck, Manjusri Das, Arthur W. Lian, Alo Ray, Reena Shakya, Sue E. Knoblaugh, Cynthia D. Timmers, Michael C. Ostrowski, Arnab Chakravarti, Gina M. Sizemore, Steven T. Sizemore

**Affiliations:** 1 Arthur G. James Comprehensive Cancer Center, The Ohio State University, Columbus, OH 43210 USA; 2 Department of Radiation Oncology, The Ohio State University, 646A TMRF, 420 W. 12th Avenue, Columbus, OH 43210 USA; 3 Target Validation Shared Resource, The Ohio State University, Columbus, OH 43210 USA; 4 Department of Veterinary Biosciences, The Ohio State University, Columbus, OH 43210 USA; 5 The Hollings Cancer Center, Medical University of South Carolina, Charleston, SC 29425 USA; 6 Division of Hematology and Oncology, Medical University of South Carolina, Charleston, SC 29425 USA; 7 Department of Biochemistry and Molecular Biology, Medical University of South Carolina, Charleston, SC 29425 USA

**Keywords:** Breast cancer, Triple-negative breast cancer, RALA, RALB, Metastasis, Small GTPases, Ral inhibitors, BQU57

## Abstract

**Background:**

Breast cancer (BC) is the most common cancer in women and the leading cause of cancer-associated mortality in women. In particular, triple-negative BC (TNBC) has the highest rate of mortality due in large part to the lack of targeted treatment options for this subtype. Thus, there is an urgent need to identify new molecular targets for TNBC treatment. RALA and RALB are small GTPases implicated in growth and metastasis of a variety of cancers, although little is known of their roles in BC.

**Methods:**

The necessity of RALA and RALB for TNBC tumor growth and metastasis were evaluated in vivo using orthotopic and tail-vein models. In vitro, 2D and 3D cell culture methods were used to evaluate the contributions of RALA and RALB during TNBC cell migration, invasion, and viability. The association between TNBC patient outcome and RALA and RALB expression was examined using publicly available gene expression data and patient tissue microarrays. Finally, small molecule inhibition of RALA and RALB was evaluated as a potential treatment strategy for TNBC in cell line and patient-derived xenograft (PDX) models.

**Results:**

Knockout or depletion of RALA inhibited orthotopic primary tumor growth, spontaneous metastasis, and experimental metastasis of TNBC cells in vivo. Conversely, knockout of RALB increased TNBC growth and metastasis. In vitro, RALA and RALB had antagonistic effects on TNBC migration, invasion, and viability with RALA generally supporting and RALB opposing these processes. In BC patient populations, elevated *RALA* but not *RALB* expression is significantly associated with poor outcome across all BC subtypes and specifically within TNBC patient cohorts. Immunohistochemical staining for RALA in patient cohorts confirmed the prognostic significance of RALA within the general BC population and the TNBC population specifically. BQU57, a small molecule inhibitor of RALA and RALB, decreased TNBC cell line viability, sensitized cells to paclitaxel in vitro and decreased tumor growth and metastasis in TNBC cell line and PDX models in vivo.

**Conclusions:**

Together, these data demonstrate important but paradoxical roles for RALA and RALB in the pathogenesis of TNBC and advocate further investigation of RALA as a target for the precise treatment of metastatic TNBC.

**Supplementary Information:**

The online version contains supplementary material available at 10.1186/s13058-021-01438-3.

## Background

Breast cancer (BC) is the most common cancer in women, and despite improvements in the diagnosis and treatment of these women, BC recently became the leading cause of cancer-associated mortality in women world-wide [1]. While patients with estrogen receptor (ER-positive) and human epidermal growth factor receptor 2 (HER2-positive) BC benefit from targeted therapies, treatment options for women with triple-negative breast cancer (TNBC) have changed little over the last few decades. While the combination of atezolizumab and nab-paclitaxel was recently approved for TNBC patients with advanced, unresectable metastatic lesions expressing programmed death ligand-1 (PD-L1) [2, 3]; untargeted, toxic chemotherapy remains the only systemic treatment option to reduce risk of recurrence for women with early stage TNBC and the only option for advanced TNBC which does not respond to PD-L1 targeting therapies [4]. Thus, there is an urgent need to identify new molecular targets and innovative treatment strategies to improve outcomes for women with TNBC.

RALA and RALB are highly homologous small G-proteins belonging to the Ras superfamily of small G-proteins. The RALs switch between inactive GDP-bound and active GTP-bound states and thus function as a molecular switch within key signaling pathways [5, 6]. RAL GTP binding and hydrolysis are regulated by common sets of guanine nucleotide exchange factors (RALGEFs) including RALGDS and RGL1-3 and GTPase-activating enzymes (RALGAPs) such as RALGAP1 and RALGAP2 [5, 6]. The RALs are best known as downstream effectors of RAS, which activates the RALGEFS [7]. The RALs, in turn, influence cell biology through interactions with a number of effector molecules including SEC5 (EXOC2) and EXO84 (EXOC8), two members of the exocyst complex, and RAL-binding protein 1 (RALBP1), which regulates a diverse set of cellular activities including CDC42 and RAC1 activity, receptor endocytosis [8–10], and mitochondrial fission [11, 12]. The RAL isoforms have also been reported to interact with additional effector molecules such as phospholipase D1, filamin, and PLCδ1 [5, 6]. As with the RALGAPs and RALGEFs, there appears to be no clear specificity among the downstream effectors for either RAL isoform [5, 6].

Despite the striking similarities between RALA and RALB protein structure, overlapping GAP and GEF utilization, and a shared pool of downstream effectors, the RAL isoforms demonstrate unexpected disparity, even antagonism, of function in various cancers [5, 13]. In non-small cell lung cancer (NSCLC), RALA and B have complementary or redundant roles. In cell line models of NSCLC, genetic knockdown of either isoform inhibited cell growth in vivo [14]. In a genetically engineered mouse model of lung adenocarcinoma, knockout of both isoforms was required to decrease tumor growth [15]. However, the similarity of RALA and B function in lung cancer appears to be the exception rather than the rule. In pancreatic cancer, RALA and B have discrete roles with RALA supporting anchorage independent growth and tumor growth, while RALB is required for invasion and lung colonization [16]. In the bladder cancer cell line UMUC-3, RALA inhibits motility while RALB promotes movement [17]. Likewise, in colorectal cancer cell lines, RALA and RALB have antagonistic functions with silencing of RALA decreasing anchorage independent growth while silencing RALB has the opposite effect [18]. The requirement of the RALs during normal mammalian development also differs with homozygous loss of *Rala* proving embryonically lethal in mice while *Ralb* null mice are viable with no overt phenotype [15]. The inconsistency of RAL function across various cancer types illustrates the critical need to thoroughly evaluate their individual contributions in any cancer in which they may contribute to disease progression.

To date, investigation of the RALs in cancer has been understandably focused upon malignancies with higher rates of RAS mutation such as lung and pancreatic cancers. Currently, less is known of the contribution of the RALs in BC [5, 6]. While RAS mutation is rare in BC, the RAS-RALGEF-RAL pathway propagates aggressive cancer phenotypes downstream of activated receptor tyrosine kinases such as epidermal growth factor receptor (EGFR) which is frequently overexpressed in TNBC [19]. The need to evaluate roles for the RALs in TNBC is particularly important as these small G proteins are emerging as potential therapeutic targets [6]. The goal of the current work was to evaluate the clinical relevancy of RAL isoforms in TNBC and gain insight into their suitability as targets for novel TNBC treatments.

We demonstrate, using 2D and 3D in vitro methods as well as orthotopic and tail-vein injection in vivo models, a clear necessity for RALA in maintaining aggressive tumor phenotypes and promoting tumor growth and metastasis in TNBC cell line models. While the role of RALB in TNBC is slightly less clear, based on our findings, RALB appears to predominantly inhibit TNBC tumor growth and progression. CRISPR-mediated knockout of RALB significantly increased orthotopic TNBC tumor growth in vivo, increased 3D invasion, and increased cell viability in both adherent and nonadherent culture conditions. Our findings are dissimilar from those reported in three recent studies where RALA and RALB were found to play similar roles in BC cell line models [20, 21] or transient silencing of RALB was found to decrease BC cell line invasion [22]. In silico analyses utilizing the large TCGA and METABRIC breast cancer patient gene expression databases uncovered paradoxical associations between survival and *RALA* and *RALB* expression in support of our in vitro and in vivo findings. These results are further supported by immunohistochemical (IHC) analyses of RALA protein expression in a BC patient cohort comprised of all BC subtypes and a separate cohort of only TNBC samples. In both cohorts, RALA IHC staining is significantly prognostic of poor outcome. Despite the contradictory associations of RALA and RALB with TNBC cell growth in vitro or in vivo, we demonstrate that BQU57, a small molecule inhibitor of both RAL isoforms, reduces TNBC cell viability, increases sensitivity to chemotherapy and hinders orthotopic tumor growth and metastasis of a TNBC cell line and patient-derived xenograft model in vivo. This suggests RALA’s tumor and metastasis promoting functions predominate RALB’s tumor and metastasis-inhibiting activities. Thus, RALA and RALB appear to have critical but opposing effects in TNBC. Importantly, while both RALs have potential as prognostic biomarkers, targeting RALA in particular has significant therapeutic potential in BC in general and TNBC in particular.

## Methods

### Cell lines

MDA-MB-231 and MDA-MB-468 human breast carcinoma cell lines were acquired from ATCC and cultured in RPMI 1640 medium. MVT1 mouse mammary tumor cells have been described previously [23] and were grown in DMEM medium. Media contained 10% fetal bovine serum (FBS), 2% pen strep, 1% l-glutamine. Cells were kept at 37°C with 5% CO_2_.

Small interfering RNA (siRNA)-mediated knockdown of RALA and RALB was achieved through the transfection of MDA-MB-231 or MDA-MB-468 cells with 50 pMol of siRNA targeting human RALA (GCAGACAGCUAUCGGAAGA; Dharmacon, Lafayette, CO, USA), RALB (GAAAGAUGUUGCUUACUAU, Dharmacon) or simultaneously targeting both isoforms (GAGCUAAUGUUGACAAGGU; Dharmacon) or non-targeting control siRNA pool (D-001810-10-05, Dharmacon) using Lipofectamine RNAiMAX (Invitrogen, Carlsbad, CA, USA) for 72 h.

Human *RALA* (TL309957V) and *RALB* (TL309956V) targeting shRNA lentiviral particles and a non-targeting control (TR30021V) were purchased from OriGene (Rockville, MD, USA). MDA-MB-231 cells were transduced with lentiviral particles and selected using 5 μg/mL puromycin for > 7 days. Similarly, mouse *RALA* targeting shRNA lentiviral particles and a non-targeting control (sc-41843; Santa Cruz Biotechnology, Inc., Dallas, TX, USA) were used to achieve knockdown of RalA in MVT1 cells. Again, cells underwent selection in puromycin-containing medium for > 7 days.

### CRISPR/Cas9 gene editing

For CRISPR-mediated knockout of RALA or RALB in MDA-MB-231 cells, paired gRNAs were designed using ATUM CRISPR gRNA design tool (atum.bio; Newark, CA, USA). Sequences for used to target RALA are 5′-aaa gtc atc atg gtg ggc ag-3′ and 5′-gcc aaa gaa ttc tga ccc tt-3′ and for RALB the sequences: ′-atg gtt ggc agc gga ggc gt-3′ and 5′-tgt gga ggg cca agg agc tc-3′ were used. Following gRNA design, a customized all-in-one vector (pSpCas9n-BB-2A-Puro (PX459) v2.0) was generated by GenScript (Piscataway, NJ, USA). Plasmids were transfected into MDA-MB-231 cells using FuGENE HD Transfection Reagent (Promega, Madison, WI, USA). Cells were selected in puromycin-containing media for >7 days. Single-cell colonies were chosen following additional sub-culturing in puromycin-containing media and were evaluated for RalA knockout by western blot analysis. Three to five individual clones with confirmed knockout were combined as a pool for further study.

### Immunoblots

See Sizemore et al. [24] for western blotting and immunodetection protocol. Antibodies were obtained from the Cell Signaling Technologies (RalA: 4799, RalB: 90879, β-actin: A1978, anti-mouse-IgG HRP: 7076, anti-rabbit IgG HRP: 7074) and Millipore Sigma (GAPDH: G8795 or MAB374).

### Patient datasets

Breast cancer cell line gene expression data was obtained from the Broad Institute Cancer Cell Line Encyclopedia (CCLE, [25]). METABRIC [26] patient mRNA data was obtained through Oncomine. TCGA patient mRNA and protein data either came from Oncomine (www.oncomine.org) or cBioPortal (Firehose Legacy, cbioportal.org, [27, 28]). ROC Plotter (rocplot.org, [29]) was used to determine gene expression correlation with treatment response.

### Patient tissues samples and PDX model

A commercially available tissue microarray (TMA) was obtained from the US Biomax, Inc. (HBre-Duc150Sur-01) and consisted of 150 individual BC patient samples. TNBC tissue microarrays (TMA) were constructed from archived breast tumor tissue isolated from 60 patients treated at the OSU James Comprehensive Cancer Center upon informed consent following approval from The OSU Institutional Review Board (IRB). The TMAs are maintained by the Columbus Breast Cancer Tissue Bank and were described previously [30]. Use of the TMAs herein was approved under IRB protocol #2016C0025. The patient-derived xenograft model (PDX-TM00096) was derived from a TNBC lung metastasis and purchased from Jackson Laboratories and maintained by the Target Validation Shared Resource (OSUCCC).

### Animal treatments and procedures

Animal use was in compliance with the University Laboratory Animal Resources (ULAR) regulations under the OSU Institutional Animal Care and Use Committee (IACUC)–approved protocol 2007A0120-R4. Adult, female FVB/N mice were purchased from the Jackson Laboratories (Bar Harbor, Maine, USA), and nod scid γ (NSG) mice were acquired through the Target Validation Shared Resource (OSUCCC).

For orthotopic fat pad injections, 5 x 10^5^ MVT1 cells and 2.5 x 10^6^ MDA-MB-231 cells were injected into 6–8-week-old female FVB/N, or 6–8-week-old female NSG mice, respectively. For orthotopic tumor experiments utilizing MDA-MB-231 CRISRP lines, 3 x 10^6^ cells were injected into 11 week old NSG mice. Once palpable tumors were detected, two-dimensional caliper measurements of tumor size were made three times per week. The following formula was used to calculate tumor volume: volume = ½ (length x width^2^). Tumor tissue and lung tissue were collected for histological evaluation at a predetermined experimental endpoint (additional details for each cohort are provided in the text and/or figure legend). Early removal criteria (ERC) was defined as tumor diameter in excess of 1.6cm, ulceration >2mm, or body condition score <2.

Luciferase-tagged MDA-MB-231 cells were generated by transduction with EF1a-luciferase lentiviral particles (GenTarget, Inc., San Diego, CA, USA; #LVP435). Cells were selected for > 10 days in neomycin-containing media (1mg/ml) prior to use in any experiments. 5 x 10^6^ cells were injected intravenously by tail-vein into 6–8-week-old female NSG mice. Bioluminescence imaging was performed according to the protocol described by Yang et al. [31]. Mice were imaged at day 0 (i.e., within 2 h post-injection) and 2x weekly (M and Th) for long-term tumor studies and at 24, 72, and 96 h post-injection for short-term analysis. All images were acquired on the IVIS Lumina II optical imaging system (PerkinElmer Inc.) available within the OSUCCC Small Animal Imaging Core.

BQU57 (50μM) in DMSO was administered 3x weekly (M-W-F) until early removal criteria were met. DMSO was given as a vehicle control. For both control and treatment arms, body condition score was noted, and individual mouse weights were measured 3x per week as a measure of drug-related toxicity. Treatment of MDA-MB-231 tumor-bearing mice commenced 21 days following orthotopic injection of cells. For the TNBC PDX model, BQU57 treatment began 24 days after implantation when the PDX tumors were on average 100 mm^3^.

### Quantification of lung metastatic tumor burden

**Fig. 1:**
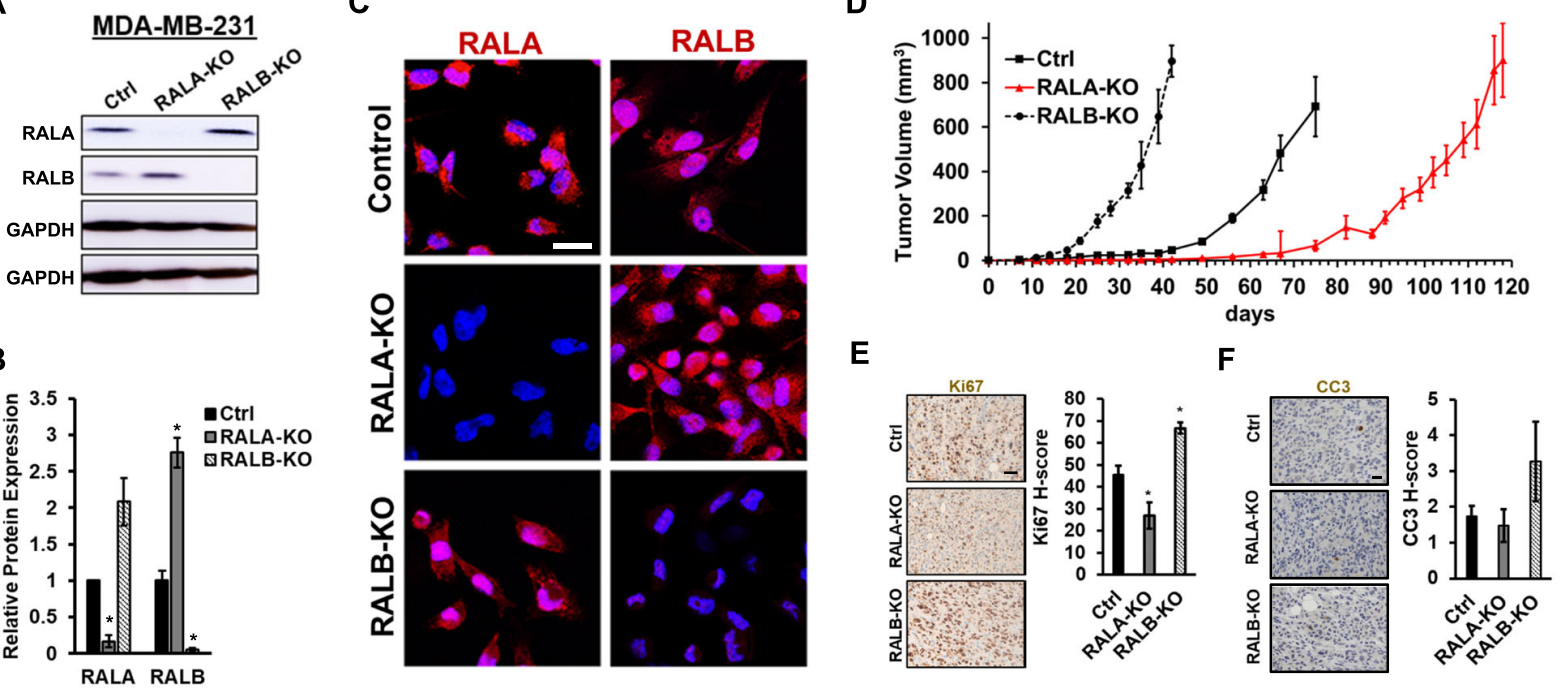
RALA, not RALB, is pro-tumorigenic in preclinical in vivo models of TNBC utilizing CRISPR mediated knockout of RALA/B. **a** Western blots demonstrating RALA and RALB expression in MDA-MB-231 CRISPR control (Ctrl), RALA CRISPR knockout (RALA-KO), and RALB CRISPR knockout (RALB-KO) cells. **b** Quantification of RALA and RALB expression in MDA-MB-231 Ctrl, RALA-KO, and RALB-KO cells by ImageJ analysis (*n* = 3). **c** Representative images of RALA and RALB immunofluorescence staining in MDA-MB-231 Ctrl, RALA-KO and RALB-KO cells (red = RALA or RALB, blue = DAPI; scale bars = 20μm). **d** Quantification of MDA-MB-231 Ctrl (*n* = 10), RALA-KO (*n* = 8), and RALB-KO (*n* = 12) orthotopic mammary tumor growth. **e** Representative images and H-score quantification of Ki67 immunostaining in MDA-MB-231 CRISPR Ctrl, RALA-KO, and RALB-KO orthotopic mammary tumors (scale bar = 40μm). **f** Representative images and H-score quantification of cleaved caspase 3 (CC3) immunostaining in MDA-MB-231 Ctrl, RALA-KO, and RALB-KO orthotopic mammary tumors (scale bar = 20μm). Data are presented as mean ± SEM; (*), *P <* 0.05

Hematoxylin and eosin (H&E)-stained lung sections were either scored for metastatic nodules, foci, and emboli visually by a veterinary pathologist (SEK) or sections were scanned on a high-resolution, Leica Aperio ScanScope XT for image analysis. The entire lung was sectioned and serial sections were taken 100μm apart. Either three sections (Fig. 3b and Supplemental Figure S1B), two sections (Fig. 6e), or one section (Fig. 1a and Supplemental Figure S1C) per mouse were used for analyses.

For image analyses of metastatic burden, whole-slide images were acquired with an Aperio XT slide scanner (Leica Biosystems, Inc., Buffalo Grove, IL, USA) using the 40x objective at The Ohio State University Comparative Pathology & Mouse Phenotyping Shared Resource. Images were imported into Visiopharm Image Analysis software (Visiopharm, Hørsholm, Denmark version 2017.27.0.3313). Images were segmented into areas of tumor metastases and normal lung tissue using different color labels for each tissue type. Tumor metastases (blue), normal tissue (green), and bronchiolar epithelium (yellow), area of red blood cells (red), and air spaces (pink) labels. A custom algorithm/App was written using a Visiopharm Decision Forest classification algorithm set at an accuracy of 50 (range 0–100). To account for size variance and metastases too small to detect, metastases measuring 8500μm^2^ and above were labeled and measured as metastases. Small misshaped areas and small metastatic areas under 8500μm^2^ were included in the normal tissue quantification. Tissue types were established, and mark-ups reviewed in consultation with a veterinary pathologist board-certified by the American College of Veterinary Pathologists (SEK) to ensure accurate measurements and differentiate between tissue types. These data were used to calculate average number of metastasis per section (total lung area), the number metastatic lesions per section (total lung area), and the percentage of total lung area occupied by metastasis as indicated.

### Immunostaining and quantification

For immunofluorescence of MDA-MB-231 CRISPR variants, 25,000 cells were seeded on an 8-well chamber slide (Corning, 354118). Cells were fixed in 4% paraformaldehyde, permeabilized in 0.5% Triton X-100, and then blocked in a 5% BSA-0.3% Triton x-100 solution. RalA (Abcam, 1:200, ab126627) or RalB (Abcam, 1:200, ab223479) primary antibody were diluted in blocking buffer and applied overnight at 4°C. Alexafluor 594 anti-rabbit secondary antibody (Invitrogen, 1:250, A21207) was added for 1 h at room temperature before removing chamber slides and coverslipping with Prolong-Gold Antifade Reagent with DAPI (Invitrogen, P36931). Images were taken on a Zeiss LSM 800 confocal microscope using the Zen software (Carl Zeiss Microscopy, LLC, White Plains, NY, USA).

Immunohistochemistry (IHC) on BC patient TMAs was done using the Bond RX autostainer (Leica Biosystems, Inc.). Briefly, slides were baked at 65°C for 15 min and the automated system performed dewaxing, rehydration, antigen retrieval, blocking, primary antibody incubation with α-RalA (1:2000, #ab126627, Abcam), post primary antibody incubation, detection (DAB), and counterstaining. Samples were then removed from the machine, dehydrated and mounted.

Ki67 and Cleaved Caspase-3 [α-Ki67 (1:100; ab16667, Abcam); α-Cleaved Caspase-3 (Asp175); 9661, Cell Signaling Technologies] immunostaining was done as previously described [32]. Sections were deparaffinized in xylenes, rehydrated, and antigen retrieval was performed using an EDTA Decloaker (Biocare Medical, LLC, Pacheco, CA, USA) for 40 min in a steamer (90°C). Hydrogen peroxide (3%) was used to quench endogenous peroxidase before tissues were blocked with 5% BSA + 0.5% Tween-20 in PBS. Sections were incubated overnight in primary antibody. The following day a biotinylated secondary antibody was added, and a Vectastain® ABC (HRP) kit along with DAB substrate (Vector Laboratories, Inc., Burlingame, CA, USA) were used to develop signal. Tissues were then dehydrated, counterstained with hematoxylin, and coverslipped.

IHC images were taken on the PerkinElmer’s Vectra® Automatic Quantitative Pathology Imaging System. The acquisition workflow has been described [32]. Quantification and scoring of RalA immunostaining on BC patient TMAs was done using inForm® Advanced Image Analysis software (PerkinElmer). InForm® software was used to spectrally un-mix images and the DAB signal was scored based on a user-defined threshold into four categories (0+, 1+, 2+, and 3+). The percent of cells within each scoring category was determined based on cell segmentation with the hematoxylin counterstain. An H-Score was calculated by the following formula: [1x(%cells 1+) + 2x(%cells 2+) + 3x(%cells 3+)]. Quantification of Ki67 and Cleaved Caspase-3 (CC3) (all but CRISPR experiments) was performed similarly using inForm® software (PerkinElmer). Quantification of CC3 for MDA-MB-231 CRISPR Ctrl and KO cell lines was performed on ImageJ Fiji Software by a previously described method [33, 34]. Briefly, images were taken on an EVOS M7000 Imaging System (ThermoFisher Scientific, Waltham, MA, USA). Color deconvolution and threshold ranges for DAB and hematoxylin staining were set to the same range (user defined) for each image. DAB signal and cell count was then calculated. DAB intensity was normalized by the number of nuclei in each image.

### Invasion, migration, and wound healing assays

Cells were serum-starved overnight prior to invasion and migration assays. Invasion assays were performed using 24-well Corning Matrigel Matrix chambers with 8-μm pore size (Fisher Scientific). Costar 24-well, 8-μm polycarbonate pore membrane, 6.5-mm inserts were utilized for migration assays (Fisher Scientific). Cells were trypsinized and 50,000 viable cells were seeded to the upper chamber. The bottom chambers held serum-containing medium to serve as a chemoattractant. Migration was allowed to proceed for 6 at 37°C with 5% CO_2_, while invasion was assessed after 24 h. After the designated incubation period, all non-migrated or non-invaded cells were removed using a cotton swab. Cells were then fixed in methyl alcohol with 1.8 mg/L of Triarylmethane Dye and stained with 1.25 g/L Thiazine Dye Mixture (Dade Behring, Deerfield, IL, USA). Invaded or migrated cells were quantified using ImageJ (National Institute of Health, Bethesda, MD, USA) [35].

Confluent plates of cells were serum-starved overnight before wound healing assay. Plates were scratched with a p200 pipette tip then washed with PBS and replenished with serum-free media. Cell migration was imaged at 0 and 6 h at 4X magnification on an EVOS XL Core Imager. Data was quantified by ImageJ software.

### GILA and soft agar assays

Growth in low attachment (GILA) was measured using the CellTiter-Glo luminescent assay (Promega, G7570). 2000 MDA-MB-231 and MDA-MB-468, or 10,000 MVT1 cell variants were plated in Ultra Low Attachment polystyrene 96-well plates (7007; Corning, Corning, NY, USA) for 5 days. A 100-μL CellTiter-Glo was added and the cells were shaken for 5 min. Following a 25-min incubation, the cells were moved to a white polystyrene a 96-well plate (3610; Corning) to read luminescence signal using a Promega Glomax Discover microplate reader. For DMSO and BQU57 treatment studies, the drug was added to growth media at day = 0.

For soft agar assays, 0.6% agar was layered in a 96-well plate. Cells were suspended in 0.4% agar at 10,000 cells/well. Each well was topped with 100μL of media. For DMSO or BQU57 treatment, media contained drugs at a 50-μM final concentration. After incubation for 7 days at 37°C with 5% CO_2,_ anchorage-independent growth was determined using CytoSelect 96-well Cell Transformation Assay, Soft Agar Colony Formation Kit (Cell Biolabs, Inc., San Diego, CA, USA).

### Spheroid assays

Spheroids were generated using a protocol adapted from Andersen et al. 2016 and Trevigen’s protocol outlined in their Spheroid Formation Kit (Trevigen; Cat#: 3511-096-K). In brief, 10,000 MDA-231 cells in 50uL of DMEM with Trevigen Spheroid Formation Matrix (Trevigen Cat#: 3500-096-SP) were seeded in 96-well, ultra-low attachment (ULA) round bottom plates (Corning Cat# CLS7007-24EA). ULA-round bottom plates were centrifuged at 750g’s for 10 min at 4*C, and incubated at 37*C at 5% CO_2_ until ready to use. Spheroids used for invasion assays were used between 7 and 12 days old.

For spheroid invasion assays, p10 tips were cut with a sterile #10 blade under tissue culture conditions. A 10uL of media with a single spheroid was transferred into a new 96-well, ULA round bottom plate. A 50uL of thawed Cultrex Basement Membrane Extract (BME) (Trevigen; Cat# 3432-001-01) was carefully added with ice-cold tips to each well. Plates were centrifuged at 300g’s for 5 min at 4*C to center the spheroids. An additional 300g’s for a 5-min centrifugation step was preformed if the spheroids were not positioned correctly. Plates were incubated at 37*C at 5% CO_2_ for 1 h prior to the careful, dropwise addition of 100uL of pre-warmed DMEM as to not dislodge the spheroids. Spheroids were cultured for 5 days and then imaged using the EVOS Core XL microscope at the 4X objective daily and were analyzed using the ImageJ/Fiji software (Schindelin et al. 2012).

### Viability, proliferation, and apoptosis assays

For determining cell viability, 50,000 MDA-MB-231 cell variants were plated into a 6-well plate. At 72 h, cells were trypsinized, harvested, stained with trypan blue, and counted using a hemocytometer. Cells were deemed viable upon trypan blue exclusion. The Cell Proliferation Kit I MTT Assay (Millipore Sigma, St. Louis, MO, USA) was also used to evaluate tumor cell viability over time. 2000 MDA-MB-231, MDA-MB-468, or MVT1 variants were plated and measurements taken at 72 h using a Promega Glomax Discover microplate reader. For DMSO, BQU57, and Taxol treatments, the media contained drugs at the indicated concentration and measurements were taken after 72 h.

The BrdU Cell Proliferation Assay (Cell Signaling, Danvers, MA, USA) was used to evaluate tumor cell proliferation. 10,000 MDA-MB-231 or 5000 MVT1 cell variants were plated and the assay was performed following manufacturer’s instructions. At 72 h, absorbance was read at 450 nm using a Promega Glomax Discover microplate reader.

The Dead Cell Apoptosis Kit with Annexin V Alexa Fluor™ 488 (Invitrogen, Carlsbad, CA, USA) was used to evaluate apoptotic populations. MDA-MB-231 or MVT1 cell variants were plated and allowed to reach ~90% confluency. Floating and harvested cells were collected and treated according to manufacturer’s instructions. Cells were acquired using a LSR II.

### Statistics

Statistical analyses were conducted with GraphPad Prism 7. Sample size was not predetermined statistically. Mice were randomly allocated for treatment studies. Investigators were not blinded to group allocation but were blinded to downstream analyses. For all data, normality was checked by Kolmogorov-Smirnov normality testing. Comparison between two groups of normally distributed data was done by homoscedastic or heteroscedastic unpaired two-tailed Student’s t test as appropriate. For data not normally distributed, statistical comparisons were done by the Mann-Whitney U test. Statistical significance of Kaplan-Meier survival curves was determined using log-rank. Statistical significance was established at *P* ≤ 0.05.

## Results

### CRISPR-mediated knockout of RALA inhibits while RALB knockout accelerates orthotopic tumor growth of TNBC cell lines

**Fig. 2:**
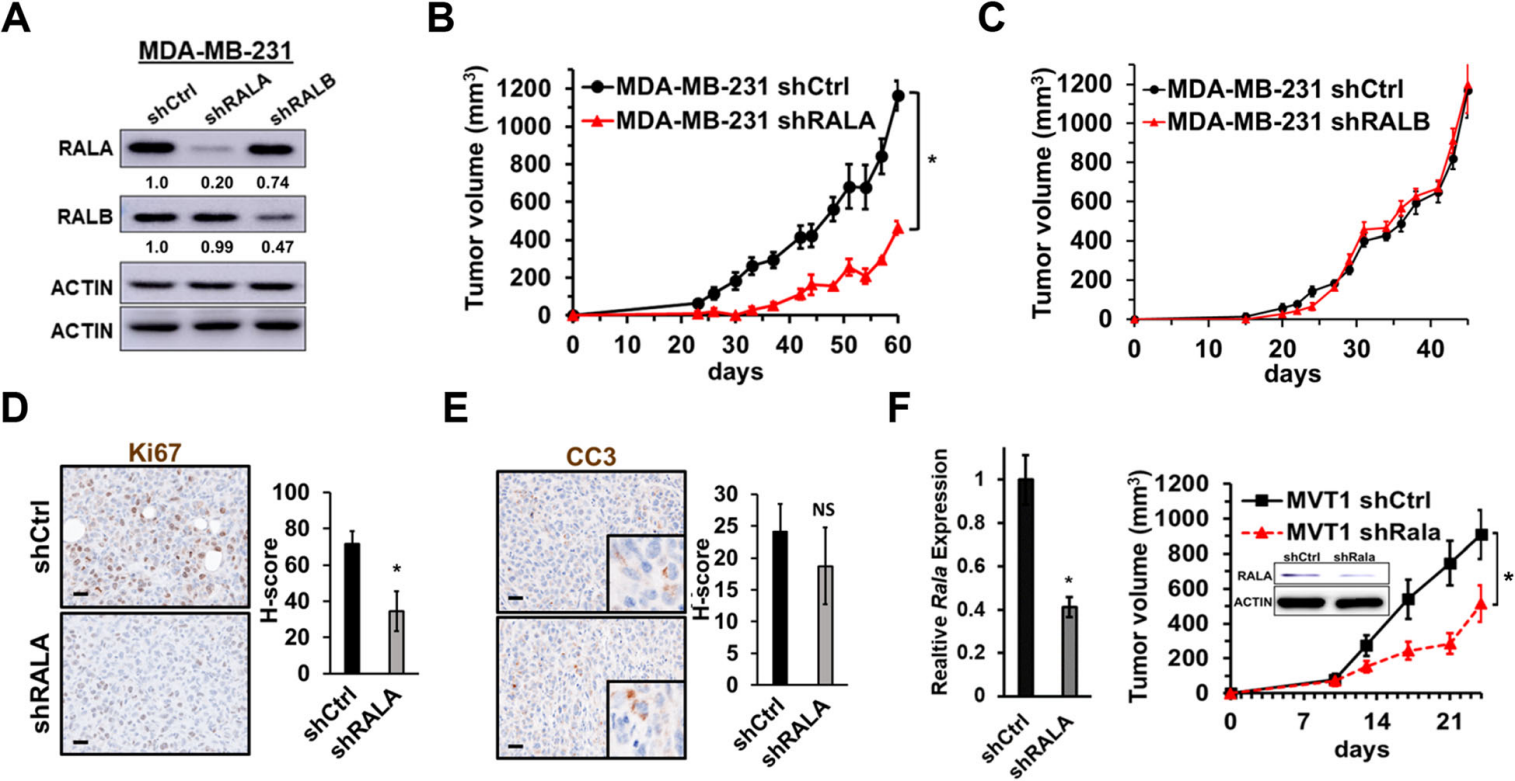
RALA, not RALB, is pro-tumorigenic in preclinical *in vivo* models of TNBC utilizing short hairpin knockdown of RALA/B. **a** Western blots demonstrating RALA and RALB expression in MDA-MB-231 shRNA control (shCtrl), shRALA, and shRALB cells. **b** Comparison of MDA-MB-231 shCtrl (*n* = 5) and shRALA (*n* = 5) orthotopic mammary tumor growth. **c** Comparison of MDA-MB-231 shCtrl (*n* = 7) and shRALB (*n* = 8) orthotopic mammary tumor growth. **d** Representative Ki67 immunostaining images and H-score quantification of MDA-MB-231 shCtrl (*n* = 4) and shRALA (*n* = 4) orthotopic mammary tumors. **e** Representative cleaved caspase 3 (CC3) immunostaining images and H-score quantification of MDA-MB-231 shCtrl (*n* = 4) and shRALA (*n* = 4) orthotopic mammary tumors. **f** Quantification of *Rala* mRNA expression in MVT1 shCtrl and shRALA cells by qRT-PCR (left) and growth of orthotopic mammary tumors (right; *n* =6 per group). Inset shows western blot confirmation of RALA protein expression in MVT1 shCtrl and shRALA cells. Data are presented as mean ± SEM; (*), *P <* 0.05; scale bars = 20μm

To begin to evaluate the clinical significance of the RAL isoforms in TNBC, we used CRISPR to knockout out each isoform individually in the human TNBC cell line MDA-MB-231 (Fig. 1a). Stable RALA knockout (RALA-KO) resulted in a significant upregulation of RALB (~3-fold, *P* < 0.05) while RALB knockout (RALB-KO) was met with a noticeable but not significant (~2-fold, *P* = 0.08) increase in RALA expression (Fig. 1b). Fluorescent IHC confirmed isoform specific knockout of each isoform and revealed a similar subcellular distribution for RALA and RALB, which was unaltered following knockout of the other isoform (Fig. 1c). Following orthotopic implantation into the mammary fat pads of NSG mice, MDA-MB-231 RALA-KO cells exhibited significantly reduced capacity to form tumors compared to control (Ctrl) cells while mice injected with MDA-MB-231 RALB-KO cells developed rapidly growing tumors (Fig. 1d). The respective cohorts were sacrificed as early removal criteria (ERC) was met. The time to ERC was 42 days for the RALB-KO group, 75 days for the Ctrl cohort, and 118 days for mice injected with RALA-KO cells. IHC staining for the proliferation marker Ki67 revealed a significant decrease in proliferation in RALA-KO tumors relative to controls while RALB-KO tumors proliferated an increased rate (Fig. 1e). IHC staining of Ctrl, RALA-KO, and RALB-KO tumor sections for the apoptosis marker cleaved caspase 3 (CC3) revealed no significant changes across groups (Fig. 1f). In additional studies, RALA and RALB were stably knocked down in MDA-MB-231 cells using shRNAs specific for each isoform (Fig. 2a). shRNA depletion of RALA (shRALA) significantly reduced the ability of MDA-MB-231 cells to growth as orthotopic tumors in NSG mice (Fig. 2b). Conversely, MDA-MB-231 cells in which RALB was stably knocked down (shRALB) grew as orthotopic tumors at a similar rate to control cells (Fig. 2c). It should be noted that shRNA depletion of RALB only reduced RALB expression to approximately 47% of control (Fig. 2a), while CRISPR knockout eliminates RALB expression (Fig. 1a, b). Furthermore, RALA expression was decreased slightly in shRALB cells (Fig. 2a) unlike in RALB-KO CRISR cells where RALA was significantly upregulated (Fig. 1a, b). These differences may explain why RALB depletion did not accelerate growth of MDA-MB-231 tumors in a similar manner to CRISPR knockout of RALB. IHC staining revealed that proliferation, as determined by Ki67 staining, was decreased in shRALA tumors relative to controls (Fig. 2d) while apoptosis measured by CC3 staining was unchanged relative to control (Fig. 2e). Finally, we tested the requirement of RALA for tumor growth in an immunocompetent mouse model. RALA was stably knocked down by shRNA in MVT1 cells (Fig. 2f, left panel and inset), a murine TNBC mammary tumor model derived from an *MMTV-cMyc/Vegfa* mouse mammary tumor [23]. Similar to the results observed in MDA-MB-231 tumor models, depletion of RALA reduced MVT1 orthotopic tumor growth in FVB/NJ mice relative to control MVT1 cells (Fig. 2f, right panel).

### RALA promotes while RALB inhibits metastatic growth of TNBC cell lines

**Fig. 3:**
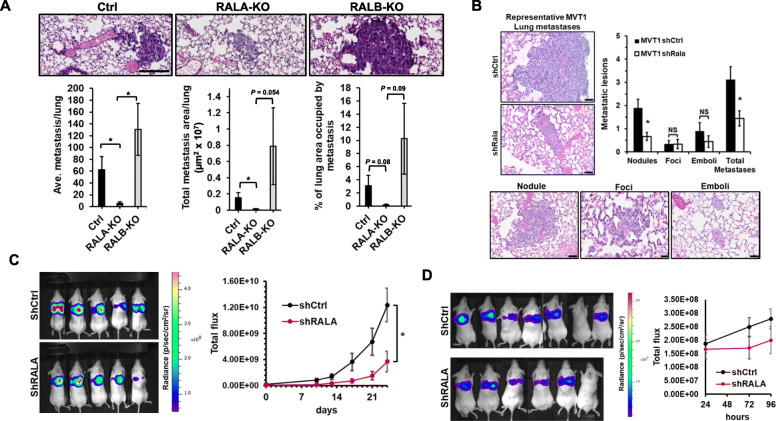
RALA, but not RALB, promotes metastatic growth in spontaneous and experimental models of TNBC lung metastasis. **a** Representative images (top) and quantitation (bottom) of lungs harvested from mice bearing MDA-MB-231 Ctrl (*n* = 5), RALA-KO (*n* = 6), or RALB-KO (*n* = 6) tumors. Lungs were harvested when early removal criteria (ERC) was reached for each respective cohort. Scale bar is 100μm. **b** Representative H&E images and quantification of lung metastases arising in mice bearing orthotopic MVT1 shCtrl (*n* = 6) or shRALA (*n* = 6) mammary tumors. Lung metastasis were categorized as nodules, foci, or emboli. Representative images of each category are shown. Scale bars are 50μm except for the representative foci image which is 20μm. All mice were sacrificed when the shCtrl group met ERC. **c** Experimental metastasis assay. Representative IVIS images and quantification of total flux in the lungs of mice following tail vein inoculation of luciferase-tagged MDA-MB-231 shCtrl (*n* = 15) or shRALA (*n* = 10) cells over 24 days. **e** Extravasation and early colonization assay. Representative IVIS images and quantification of total flux in the lungs of mice following tail vein inoculation of luciferase-tagged MDA-MB-231 shCtrl (*n* = 13) or shRALA (*n* = 11) cells over 96 h. Data are presented as mean ± SEM; (*), *P <* 0.05

We next examined the impact of RALA or RALB knockdown on spontaneous lung metastasis of MDA-MB-231 cells. Lung sections from mice bearing MDA-MB-231 control, RALA-KO, and RALB-KO tumors were analyzed for metastatic burden (Fig. 3a, and Supplemental Figure S1A). Overall, there was significant variability in lung metastases in the control and RALB-KO groups while the RALA-KO group had uniformly low levels of lung metastases. The average number of metastasis per lung as well as the total metastatic area per lung was significantly decreased in mice bearing RALA-KO tumors relative to mice with control or RALB-KO tumors (Fig. 3a, bottom left and middle panels). There was also a strong trend toward a decrease in the percentage of lung area occupied by metastasis in mice bearing RALA–KO tumors compared to the other two groups (Fig. 3a, bottom right panel). While there were no statistically significant differences in lung metastases in mice bearing RALB-KO tumors compared to the other groups due to the aforementioned, there was an obvious qualitative increase in tumor burden in the lungs of these mice compared to the other groups. Average primary tumors size at the time of sacrifice was 726.0 ± 136.3 mm^3^ for the control group, 901.27 ± 182.0mm^3^ for the RALA-KO group, 896.9 ± 71.60mm^3^ for the RALB-KO group. There was no statistical difference in primary tumor volume between the groups at time of harvest (ANOVA *P* = 0.4856). Thus, any differences in lung metastatic burden between groups cannot be attributed simply to differences in tumor size. We next examined spontaneous metastasis in FVB/NJ mice bearing either MVT1 shCtrl or shRALA tumors (Fig. 2f). The metastatic lung burden in this model is low enough to allow for detailed pathological examination and quantification of small metastatic foci (<50 cells), larger metastatic nodules, and tumor cell emboli. Depletion of RALA significantly reduced the number of large nodules, but did not reduce the incidence of small metastatic foci or tumor cell emboli in the lungs of these mice (Fig. 3b). Spontaneous lung metastasis was also analyzed in mice bearing MDA-MB-231 control, shRALA, or shRALB tumors. Depletion of RALA again was found to decrease metastatic burden (Supplemental Figure S1B) while partial depletion of RALB by shRNA, unlike complete knockout by CRISPR, did not significantly alter metastatic burden relative control (Supplemental Figure S1B). No changes in proliferation, as determined by Ki67 IHC, were observed in the metastatic nodules from the shCtrl and shRALA groups (Supplemental Figure S1D).

We further examined the necessity of RALA in metastasis and lung colonization using a tail-vein model of experimental lung metastasis. Luciferase-tagged MDA-MB-231 shCtrl or shRALA cells were injected into the tail vein of NSG mice and luminescence in the lung was measured over time until several animals from the cohort reached ERC. The luminescence signal in the lungs was significantly reduced in mice injected with shRALA cells relative to those injected with control cells (Fig. 3c). Examination of lung luminescence over a shorter time period (96 h) showed no difference in luminescence signal in the lungs between control and shRALA groups within the first few days post injection (Fig. 3d).

Together, these data suggest RALA is not necessary for cancer cell extravasation into the lungs or the early steps of metastatic colonization but does promote metastatic outgrowth.

### RALA and RALB have opposing effects on in vitro measures of cancer aggressiveness

**Fig. 4:**
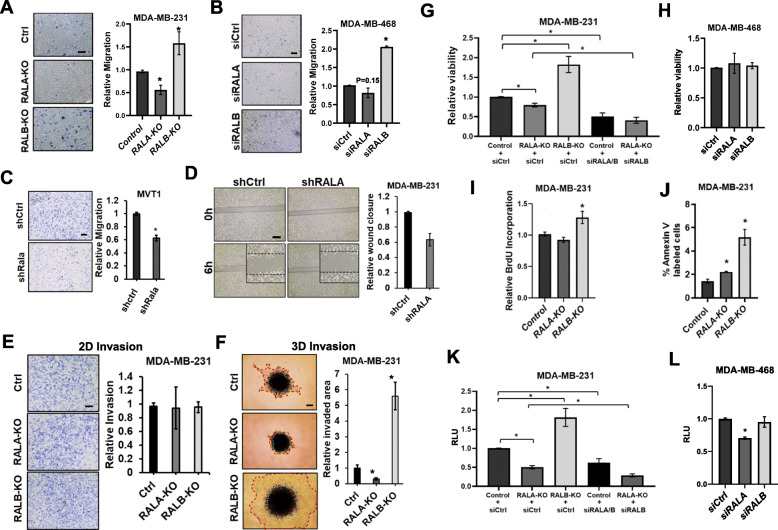
RALA supports, while RALB opposes, *in vitro* measures of aggressive cancer phenotypes in TNBC cell lines. **a** Representative images and quantification of MDA-MB-231 Ctrl, RALA-KO, and RALB-KO cell migration over 6 h (scale bar = 100μm). **b** Representative images and quantification of MDA-MB-468 siRNA control (siCtrl), siRALA, and siRALB cell migration over 6 h (scale bar = 100μm). **c** Representative images and quantification of MVT1 shCtrl and shRALA cell migration over 6 h (scale bar = 200μm). **d** Representative images and quantification of MDA-MD-231 shCtrl and shRALA scratch assay over 6 h (scale bar = 500μm). **e** Representative images and quantification of MDA-MB-231 Ctrl, RALA-KO, and RALB-KO cell invasion through matrigel coated transwell inserts over 24 h (scale bar = 200μm). **f** Representative images and quantification of MDA-MB-231 Ctrl, RALA-KO, and RALB-KO cell invasion as three dimensional spheroids over 5 days (scale bar = 400μm). **g** Quantification of viability for MDA-MB-231 Ctrl, RALA-KO, and RALB-KO cells ± siCtrl or siRALA/B transient knockdown over 72 h. **h** Quantification of viability for MDA-MB-468 siCtrl, siRALA and siRALB cells over 72 h. **i** Quantification of BrdU incorporation for MDA-MB-231 Ctrl, RALA-KO, and RALB-KO cells over 72 h. **j** Quantification of Annexin V positivity for MDA-MB-231 Ctrl, RALA-KO, and RALB-KO cells. **k** Quantification of growth in low adhesion (GILA) conditions for MDA-MB-231 Ctrl, RALA-KO, and RALB-KO cells ± siCtrl or siRALA/B transient knockdown over 5 days. **h** Quantification of growth in low adhesion (GILA) for MDA-MB-468 siCtrl, siRALA and siRALB cells over 5 days. Data are presented as mean ± SEM; (*), *P <* 0.05

Transwell migration assays were used to measure the relative migration of MDA-MB-231 control, RALA-KO, and RALB-KO cells. RALA-KO significantly reduced migration while RALB-KO increased migration relative to controls (Fig. 4a). In MDA-MB-468 cells in which RALA or RALB were transiently knocked down by siRNA (Supplemental Figure S2A), partial depletion of RALA resulted in a non-significant trend toward decreased migration while silencing of RALB significantly increased migration (Fig. 4b). Stable knockdown of RALA in MVT1 cells also decreased the migration of these cells relative to controls (Fig. 4c). Unexpectedly, when migration of MDA-MB-231 shCtrl, shRALA, and shRALB cells were compared, depletion of either RALA or RALB resulted in decreased migration (Supplemental Figure S2B). Reduced migration of MDA-MB-231 shRALA cells relative to controls was further confirmed by a wound healing assay (Fig. 4d). We next examined the relative invasiveness of control, RALA-KO, and RALB-KO MDA-MB-231 cells using transwell inserts which included a top layer of matrigel. Surprisingly, knockout of neither RALA nor RALB impacted invasion of these cells (Fig. 4e). This result was confirmed in MDA-MB-231 shCtrl, shRALA, and shRALB cells (Supplemental Figure S2C). We also analyzed invasion of MDA-MB-231 Ctrl, RALA-KO, and RALB-KO cells in a three-dimensional invasion assay. Here, cells were first grown into spheroids prior to suspension in Cultrex Basement Membrane Extract. Unlike two-dimensional invasion in transwell inserts, knockout of RALA significantly decreased, while knockout of RALB significantly increased, three-dimensional invasion relative to control cells (Fig. 4f). Based upon these results, RALA and RALB appear to have a significant, but opposing, effects upon TNBC migration and invasion and isoform specific contributions may be better assessed using more biologically relevant three-dimension assays.

Viability of MDA-MB-231 control, RALA-KO, and RALB-KO cells was measured by MTT assay. Knockout of RALA resulted in a slight but statistically significant decrease in viability while knockout of RALB significantly increased viability relative to controls (Fig. 4g). Given the opposing effects of RALA and RALB upon cell viability, we also wanted to determine the impact on viability when both RAL isoforms were depleted. To achieve this, we used siRNA to knockdown both isoforms in control cells or siRNA targeting RALB to additionally knockdown this isoform in RALA-KO cells (Supplemental Figure S2D). Interestingly, knockdown of both RAL isoforms by either method resulted in a further decrease in viability compared to control cells or RALA-KO cells (Fig. 4g). Incomplete knockdown of either RALA or RALB by shRNA was not found to impact viability in MDA-MB-231 cells (Supplemental Figure S2E). Likewise, shRNA knockdown of RALA did not alter cell viability in MVT1 cells (Supplemental Figure S2F) and transient knockdown of either RALA or RALB had no effect on viability in MDA-MB-468 cells (Fig. 4h). Changes in proliferation in MDA-MB-231 cells as a consequence of RALA or RALB loss were determined by BrdU incorporation. Knockout of RALA did not change proliferation while RALB knockout significantly increased proliferation (Fig. 4i). Similar results were seen when RALA or RALB were depleted by shRNA in either MDA-MB-231 or MVT1 cells (Supplemental Figure S2G and H). The impact of RAL isoform loss on apoptosis was measured by Annexin V labeling. In MDA-MB-231 cells, CRISPR knockout (Fig. 4j) or shRNA-mediated depletion (Supplemental Figure S2I) of either RAL isoform increased Annexin V labeling relative to control cells. Meanwhile, depletion of RALA by shRNA had no impact on Annexin V labeling of MVT1 cells relative to controls (Supplemental Figure S2J). Overall, the percentage of Annexin V labeled cells was under 5% of the total population for all conditions. Combined, these results do not make a strong case for a consistent and significant role for either RAL isoform in TNBC cell line viability, proliferation, or apoptosis during growth under adherent conditions.

We also analyzed the requirements of RALA and RALB during cell viability under low adhesion or nonadherent conditions. Either CRISPR-mediate knockout or shRNA knockdown of RALA significantly decreased MDA-MB-231 cell growth in low adhesion (GILA) conditions while RALB knockout or knockdown did the opposite (Fig. 4k and Supplemental Figure S2K). Using siRNA to knockdown both RAL isoforms in MDA-MB-231 control cells or to additionally knockdown RALB in RALA-KO cells, we found depletion of both isoforms resulted in further decreased viability relative to control or RALA-KO alone (Fig. 4k). In MDA-MB-468 cells, transient knockdown of RALA by siRNA significantly decreased cell viability under GILA conditions while silencing RALB had no effect in this cell line (Fig. 4l). In addition, knockdown of RALA in MVT cells also decreased their viability under GILA conditions (Supplemental Figure S2L). In soft agar growth assays, depletion of RALA by either CRISPR knockout or shRNA decreased nonadherent growth of MDA-MB-231 cells (Supplemental Figure S2M and N). Depletion of RALB by either CRISPR knockout or shRNA did not result in consistent changes to growth of MDA-MB-231 cells in soft agar. RALB-KO had no impact on growth (Supplemental Figure S2M) while shRNA knockdown of RALB significantly increase growth (Supplemental Figure S2N). Overall, we observed a consistent role for RALA in maintaining TNBC cell line viability under low/non-adherent conditions while RALB generally appears to play lesser, but opposing, role.

### RALA expression is associated with poor outcome in BC and specifically TNBC

**Fig. 5:**
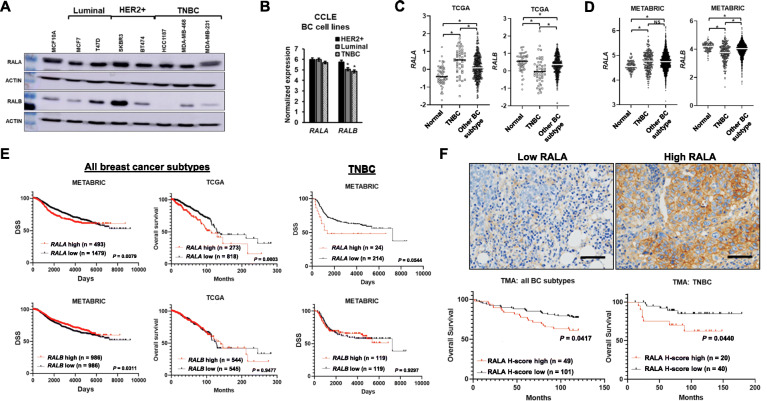
*RALA* is elevated in TNBC and is prognostic of overall survival in ER-negative disease. **a** Western blots demonstrating RALA and RALB expression in a panel of BC cell lines denoted by molecular subtype. **b** *RALA* and *RALB* mRNA expression in a panel of 46 BC cell lines categorized according to molecular subtype. Data from the Broad Institute Cancer Cell Line Encyclopedia (CCLE), (*) *P* < 0.05. **c** Box and whisker plots of *RALA* and *RALB* expression in normal breast tissue (*n* = 61), BC subtypes other than TNBC (*n* = 300) , and TNBC (*n* = 49). Data from TCGA Research Network. (*) *P* < 0.05. **d** Box and whisker plots of *RALA* and *RALB* expression in normal breast tissue (*n* = 144), BC subtypes other than TNBC (*n* = 1725), and TNBC (*n* = 250). Data from METABRIC. (*) *P* < 0.05. **e** Left: Kaplan-Meier analysis segregates all BC patients by *RALA* (upper quartile vs lower three-quartiles) and *RALB* (spilt along the median) expression in METABRIC and TCGA datasets wherein high *RALA* is prognostic of worse disease specific survival (DSS) in the METABRIC (*P* = 0.0079) and overall survival in the TCGA (*P* = 0.0003) cohorts and low *RALB* is prognostic of worse DSS in the METABRIC cohort (*P* = 0.0311), but is not prognostic in the TCGA cohort (*P* = 0.9477). Right: Within the TNBC population of the METABRIC cohort, *RALA* (upper tenth percentile vs lower 90th percentail) is prognostic of worse DSS TNBC patients (*P* = 0.0544) while *RALB* (spilt along the median) is not prognostic (*P* = 0.9297). Significance determined by log-rank. **f** Top: Representative high and low RALA immunostaining of BC patient samples. Bottom: Kaplan-Meier analysis segregating all BC patients (*P* = 0.0417) and TNBC patients (*P* = 0.0440) by RALA H-score (upper tertile vs lower two tertiles) where high RALA is prognostic of worse overall survival (Scale bars = 60μm)

To determine if RALA or RALB expression is differently associated with the various BC subtypes we performed western blot analysis on a number of cell lines representative of luminal, HER2+, and TN BC (Fig. 5a). RALA was consistently expressed at a high level in all lines and we found no association between its expression and BC subtype. RALB expression was more variable but no clear expression pattern could be ascertained from our small panel of cell lines. We next used gene expression data from the Broad Institute Cancer Cell Line Encyclopedia (CCLE) to examine *RALA* and *RALB* expression across a panel of 46 breast cancer cell lines (Fig. 5b and Supplemental Figure S3A). In this larger panel it, was clear that, while *RALA* expression does not vary across BC cell lines, *RALB* expression is significantly reduced in luminal and TN breast cancer cell lines relative to HER2+ lines (Fig. 5b). Closer examination of RAL isoform expression in only TNBC lines found no difference in expression between the more basal-like (TNA) and more mesenchymal-like (TNB) TNBC cell lines (Supplemental Figure S3B and C). To elevate any concerns protein expression may not be well correlated with mRNA expression for either RAL isoform, we examined the correlation between protein and mRNA expression in the 74 samples in the TCGA dataset for which both values were available. For both RALA and RALB, protein expression is significantly correlated with mRNA levels (Supplemental Figure S3D). Next, we analyzed *RALA* and *RALB* mRNA expression in the large TCGA and METABRIC breast cancer patient datasets. In the TCGA, *RALA* expression is significantly increased in TNBC relative to normal mammary tissue or other breast cancer subtypes (Fig. 5c, left panel) while RALB expression is significantly decreased in TNBC relative to normal mammary tissue or other breast cancer subtypes (Fig. 5c, right panel). In the METABRIC cohort, *RALA* expression is not significantly different between TNBC and other BC subtypes but is increased in BC relative to normal mammary tissue (Fig. 5d, left panel) while *RALB* expression is significantly decreased in TNBC relative to normal mammary tissue or other breast cancer subtypes (Fig. 5d, right panel).

The prognostic significance of *RALA* and *RALB* expression was next analyzed across all breast cancer subtypes in the METABRIC and TCGA datasets. High *RALA* expression (upper quartile compared to the lower three quartiles) is significantly associated with disease-specific survival in METABRIC (Fig. 5e, upper left panel). This association is quite robust and is maintained across a range of preselected segregation points (Supplemental Figure S4). Elevated *RALA* expression is also significantly prognostic of poor overall survival in the TCGA cohort (Fig. 5e, upper middle panel). Analysis of outcomes in only the TNBC patient samples of the METABRIC population revealed a strong trend toward elevated *RALA* expression and poor outcome (Fig. 5e, upper right panel) which is significant (*P* = 0.0139) when survival is analyzed only over 5 years, the period of time when most disease specific deaths occur in the TNBC group (Supplemental Figure S5). Elevated *RALB* expression, on the other hand, is significantly associated with better disease-specific survival in the METABRIC cohort when the population is segregated by median *RALB* expression (Fig. 5e, lower left panel). This association is maintained across several, but not all, preselected segregation points (Supplemental Figure S6). In the TCGA cohort, *RALB* expression was not significantly correlated with outcome at any preselected segregation point (Fig. 5e, lower middle panel and data not shown). Likewise, *RALB* is not prognostic of survival when only the TNBC population of the METABRIC cohort is considered (Fig. 5e, lower right panel).

Analysis of the prognostic significance of *SEC5/EXCO2*, *EXO84/EXOC8*, and *RALPB1,* the primary downstream effectors of RALA and RALB, found no association between their expression and outcome in the METABRIC cohort (Supplemental Figure S7). Decreased expression of *RALGAPA1*, the catalytic subunit of RALGAP1 revealed its decreased expression is associated with worse outcome in BC patients (Supplemental Figure S8A) while expression of *RALGAP2*, the catalytic subunit of RALGAP2 revealed it is not prognostic of outcome (Supplemental Figure S8B). This data implies RALGAP1 but not RALGAP2 may be critical for limiting RALA activity in BC. Interestingly, *RALGAPA1* expression is decreased in TNBC relative to normal breast tissue or other BC subtypes in the METABRIC cohort (Supplemental Figure S8C). *RALGAPA2* expression is similar between TNBC and normal breast but is elevated in other BC subtypes (Supplemental Figure S8D). Unexpectedly, elevated expression of *RALGAPB*, the non-catalytic subunit of both RALGAP1 and RALGAP2, is associated with poor outcome in the METABRIC cohort (Supplemental Figure S8E) and *RALGAPB* expression is elevated in both TNBC and other BC subtypes relative to normal breast (Supplemental Figure S8F). It is not clear how elevated expression of this common scaffold for both RALGAPs may contribute to poor outcome in BC. Expression of three RALGEFS, *RALGDS*, *RGL1*, and *RGL2*, are not prognostic of outcome (Supplemental Figure S9A-C). Elevated *RGL3* expression is associated with better outcome (Supplemental Figure S9D). *RGL3* is elevated in both TNBC and other BC subtypes relative to normal breast and is further elevated in other BC subtypes relative to TNBC (Supplemental Figure S9E).

To further evaluate RALA as a potential prognostic biomarker we utilized a commercially available tissue microarray (TMA) containing breast cancer patient samples encompassing all subtypes as well as a TMA comprised of only TNBC samples constructed at our institution (Supplemental Table 1). IHC staining for RALA was performed on both TMAs (Fig. 5f, upper panels), and high RALA expression was found to be significantly associated with poor overall survival in both cohorts (Fig. 5f, lower panels).

In total, these results suggest potential for RALA as a prognosticator of outcome in BC as a whole as well as specifically in TNBC.

### BQU57, a small molecule inhibitor of the RALs, decreases TNBC growth in vitro and in vivo

**Fig. 6:**
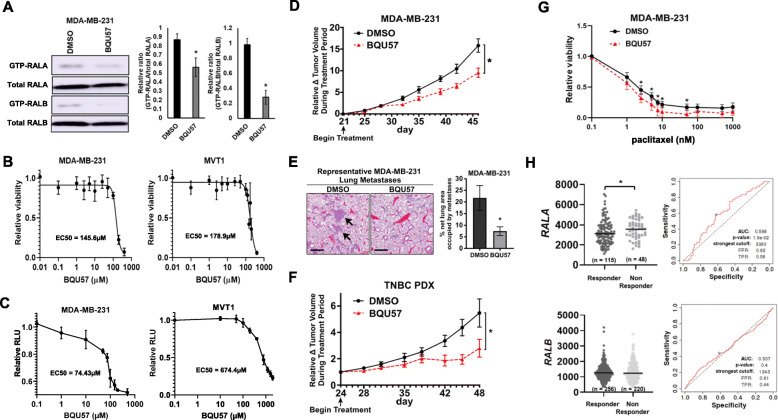
BQU57 blocks TNBC growth in vitro and in vivo. **a** Western blot and quantification of GTP-bound RALB in MDA-MB-231 cells after BQU57 (50μM) or DMSO treatment for 24 h. **b** Quantification of MDA-MB-231 and MVT1 cell viability upon treatment with varying doses of BQU57 for 72 h. **c** Quantification of MDA-MB-231 and MVT1 growth in low adhesion (GILA) conditions upon treatment with varying doses of BQU57 for 5 days. **d** Quantification of MDA-MB-231 orthotopic mammary tumor volume over time [*n* = 9 DMSO; *n* = 10 BQU57 treatment (50mg/kg by i.p. injection, M-F)]. **e** Representative H&E images (left) and quantification (right) of spontaneous lung metastases in MDA-MB-231 tumor bearing mice following treatment (arrows point to metastatic lesions). Scale bars = 500μm. **f** Quantification of subcutaneous TNBC PDX tumor volume over time. [*n* = 9 DMSO; *n* = 9 BQU57 treatment (50mg/kg, M-F)]. **g** Quantification of MDA-MB-231 cell viability upon treatment with BQU57 (100μM) or DMSO in combination with various doses of paclitaxel for 72 h. **h** RALA expression, but not RALB expression, is predictive of BC patient response to chemotherapy. Data from ROC Plotter. Data are presented as mean ± SEM; (*), *P* < 0.05

We next sought to evaluate the potential of developing RAL inhibitors as therapeutic options for TNBC. BQU57 is an experimental small molecular inhibitor of both RAL isoforms [6]. BQU57 effectively reduced activity of both RALA and RALB in MDA-MB-231 cells as determined by pulldown of the GTP-bound active isoforms (Fig. 6a). We next determined the EC50s for BQU57 in MD-MB-231, MVT, and MDA-MB-468 cells via MTT assay. BQU57 had an EC50 of 145.6μM, 178.9μM, and 486.6μM, respectively (Fig. 6b and Supplementary Figure S10A). Under low adhesion growth conditions the BQU57 EC50s for MDA-MB-231 and MVT-1 were found to be 74.43μM and 674.4μM (Fig. 6c). For MDA-MB-468, an EC50 for BQU57 could not be calculated as the limit of solubility for BQU57 was reached (~2000μM) prior to the half-maximal response (Supplemental Figure S10B).

The efficacy of BQU57 was tested in vivo by treatment of mice bearing palpable MDA-MB-231 tumors. BQU57 significantly hindered both primary tumor growth (Fig. 6d) and spontaneous lung metastasis as defined by the percent area of lung occupied by metastatic lesions (Fig. 6e). We also tested BQU57 in mice bearing patient-derived xenografts (PDX) derived from a TNBC lung metastasis. Again BQU57 significantly reduced tumor growth (Fig. 6f). Minimal toxicity, as assessed by body weight, was observed in BQU57-treated mice in either model (Supplemental Figure S10C and D).

Finally, we tested whether BQU57 could improve efficacy of a standard chemotherapeutic agent used to treat TNBC. MDA-MB-231 cells were treated with 100μM of BQU57 and a range of paclitaxel up to 1000nM. BQU57 had a significant additive effect with submaximal concentrations of paclitaxel (Fig. 6g). Furthermore, analysis of publicly available data revealed *RALA* expression, but not *RALB* expression is predictive of BC patient response to chemotherapy (Fig. 6h).

Combined, these data make a compelling case for the further development of RAL-targeting therapies as single therapy agents for the treatment of BC, particularly TNBC, as well as their use in combination with current chemotherapeutics.

## Discussion

Despite similarities between RALA and RALB in terms of protein structure, overlapping GAP and GEF utilization, and a shared pool of downstream effectors, the two protein isoforms often exhibit distinct biological functions in many cancers [5, 6]. From previous reports, one may draw the general consensus that RALA supports anchorage-independent growth whereas RALB is involved in metastasis and invasion. In fact, RALB was recently reported to be necessary for invasion in breast cancer [22]. Herein, we demonstrate that RALA drives aggressive phenotypes in TNBC while RALB appears to be less necessary, even antagonistic, for TNBC aggressiveness. In order to rectify the differences between our findings and those reported previously, and to fully understand the mechanisms of RAL-mediated BC progression, it will be important to decipher the functional differences between RALA and RALB despite their close structural homology. The first step towards achieving this understanding requires a specific elimination of one RAL isoform without off-target interference of the other isoform. Ours is the first study to analyze the separate roles of RALA and RALB in BC utilizing CRISPR gene editing to knockout each isoform individually. Targeting the RAL isoforms using RNA interference is problematic given the high similarity of these isoforms. We observed important reciprocal regulation of RAL isoform knockout that has not been reported in previous studies using interfering RNA to deplete RAL expression. Future studies will utilize our CRISPR knockout cell lines along with addback of effector-uncoupled RAL mutants to interrogate the important downstream signaling of each isoform.

Compared to the profound effect of RALA and RALB depletion on in vivo tumor growth and metastasis, several common in vitro assays, including two-dimension invasion and viability assays, showed only modest effects or results which were inconsistent with in vivo tumor growth. We observed three-dimensional invasion assays and growth under low or non-adherent conditions were better able to recapitulate the results of RAL isoform depletion in vivo. Growth in low adhesion (GILA) appears to be particularly well suited to study the different contributions of the RAL isoforms as this inexpensive and rapid assay provided a high level of concordance with in vivo results. Expression patterns of RALA and RALB were inconsistent between BC cell lines grown in vitro and BC patient samples. These results do not appear to be due to a lack of correlation between protein and mRNA expression. Instead, this may reflect a selection or adaptation mechanism involving RALA when cell lines are maintained under typical culture conditions.

Recently, the *C. elegans* RAL homolog, RAL-1, was found to be required for the biogenesis and release of extracellular vesicles, important mediators of cell-cell communication [36]. This group has also shown that shRNA knockdown of either RALA or RALB in 4T1 mammary tumor cells leads to a significant reduction in secreted exosome-like vesicles and reduced lung metastatic capacity [20, 36]. Our results suggest that RALA but not RALB contributes to metastatic outgrowth of BC, particularly TNBC, cell lines in the lungs. Additional studies will be needed determine if these disparate findings are the result of cell line-specific effects or due to differences in study design. It has also been reported [20] that elevated expression of both *RALA* and *RALB* are associated with poor outcome in the TCGA breast cancer patient cohort. Our findings confirm strong association between elevated *RALA* expression and poor outcome, but do not support a correlation between elevated *RALB* expression and reduced BC patient survival. This previous report appears to have utilized an optimize cutoff for *RALB* expression in TCGA which may over-estimate prognostic significance.

We did find not a correlation between the expression of any of the primary RAL effectors (i.e., SEC5, EXO84, RALBP1) and BC patient outcome. This is not entirely surprising as the active RAL isoforms are known to influence activity of these effectors by regulating their subcellular distribution. Our study did not directly assess RAL isoform activity or effector localization. We did find decreased expression of *RALGAPA1*, the catalytic subunit of RALGAP1, was associated with poor outcome in BC patients. This may indicate a critical role for RALGAP1 as a negative regulator of RAL activity in BC which warrants additional study.

It should also be noted that while our study focused on the roles of the RALs in TNBC, these small G-proteins are likely to be important across breast cancer subtypes. Understanding the contributions of the RALs in TNBC is particularly warranted however given the lack of therapeutic targets for this subtype and the known interconnection between RAL and EGFR signaling. EGFR is frequently over expressed in TNBC [19] and RALs are important downstream mediators of EGF signaling in both normal and malignant cells. The RALs contribute to endocytosis of EGFR [37], and RAL signaling downstream of EGF is critical for cell fate determination during development [38]. It is likely the RALs may also be particularly important mediators of tumor growth in HER2+ breast cancer as activated HER2 also activates Ras leading to RAL activation. Recently, RALA was shown to function downstream of HER2 in colorectal cancer cell lines [39], but to date, this pathway has not been explored in breast cancer.

BQU57 and its related compounds directly target both RAL isoforms non-electively which may limit their usefulness in cancers where RALA and RALB have antagonistic roles. Nonetheless, our findings indicate that even when RALA and RALB demonstrate functional antagonism in TNBC lines, simultaneous inhibition by genetic silencing or BQU57 is highly effective. In the cell lines utilized here, the RALA-silencing phenotype predominated when both isoforms were targeted. Still, development of more selective RALA inhibitors is advocated to treat cancers in which RALA is the primary driver of aggressive phenotypes. While selective RALA inhibitors are far removed from being available for clinical application, RALA can currently be preferentially targeted indirectly by Aurora-A kinase inhibitors including alisertib (MLN8267) [40]. Aurora A phosphorylates RALA on serine residues to alter subcellular localization and activity, and these target residues are not shared with RALB [41]. Importantly, alisertib is currently being evaluated for the treatment of advanced TNBC [42] and RALA expression may predict response in these patients.

## Conclusions

While both RALA and RALB have been implicated as drivers of aggressive phenotypes in many types of cancer, our study demonstrates a pro-tumorigenic and pro-metastatic role for RALA in TNBC which is not shared with RALB. This work highlights the therapeutic potential of targeting RALA for the treatment of TNBC. Indeed, we demonstrate efficacy of a RAL inhibitor in blocking in vivo orthotopic tumor growth and metastasis of a TNBC cell line as well as a patient-derived xenograft model.

## Supplementary information

**Additional file 1: Supplemental Figure S1. (A)** Representative low magnification images of lungs harvested from mice bearing MDA-MB-231 Ctrl, RALA-KO, or RALB-KO tumors. Scale bar = 2000μm. **(B and C)** Representative images and quantification of lung nodules resulting from MDA-MB-231 shCtrl (*n* = 4) and shRALA (*n* = 4) orthotopic mammary tumors (B) or shCtrl (*n* = 10) and shRALB (*n* = 12) tumors (C). Tumor-bearing mice were sacrificed when the respective shCtrl group met ERC. **(D)** Representative images and H-score quantification of Ki67 immunostaining in lung tumor nodules from mice bearing MDA-MB-231 shCtrl or shRALA tumors. Error bars represent SEM; *, *P <* 0.05. **Supplemental Figure S2. (A)** Western blots demonstrating RALA and RALB expression in MDA-MB-468 siRNA control (siCtrl), siRALA, and siRALB cells. **(B)** Representative images and quantification of MDA-MB-231 shCtrl, shRALA and shRALB cell migration after 6 h (scale bar = 200μm). **(C)** Representative images and quantification of MDA-MB-231 shCtrl, shRALA and shRALB cell invasion through matrigel coated transwell inserts after 24 h (scale bar = 200μm). **(D)** Western blots demonstrating RALA and RALB expression for MDA-MB-231 Ctrl, RALA-KO, and RALB-KO cells ± siCtrl or siRALA/B transient knockdown. **(E)** Quantification of viability for MDA-MB-231 shCtrl, shRALA, and shRALB cells. **(F)** Quantification of viability for MVT1 shCtrl and shRALA cells over 72 h. **(G)** Quantification of BrdU incorporation for MDA-MB-231 shCtrl, shRALA, and shRALB cells over 72 h. **(H)** Quantification of BrdU incorporation for MVT1 shCtrl and siRALA cells over 72 h. **(I)** Quantification of Annexin V positivity for MDA-MB-231 shCtrl, shRALA, and shRALB cells. **(J)** Quantification of Annexin V positivity for MVT1 shCtrl and siRALA cells. **(K)** Quantification of growth in low adhesion (GILA) conditions for MDA-MB-231 shCtrl, shRALA and shRALB cells for 5 days. **(L)** Quantification of growth in low adhesion (GILA) conditions for MVT1 shCtrl and shRALA cells for 5 days. **(M)** Quantification of growth in soft agar for MDA-MB-231 Ctrl, RALA-KO, and RALB-KO cells over 7 days (relative to controls). **(N)** Quantification of growth in soft agar for MDA-MB-231 shCtrl, shRALA and shRALB cells over 7 days (relative to shCtrl). Data are presented as mean ± SEM; (*), *P <* 0.05. **Supplemental Figure S3. (A)** Normalized *RALA* and *RALB* expression in breast cancer cell line data from the Broad Cancer Cell Line Encyclopedia (CCLE). **(B)** Quantification of normalized *RALA* and *RALB* expression in TNBC cell lines classified as TNBC-A (more basal-like) or TNBC-B (more mesenchymal like). Data is from CCLE**. (C)** Western blots illustrating RALA and RALB expression in representative TNBC-A and TNBC-B lines. **(D)** Correlation of mRNA and protein expression levels for RALA (top) and RALB (bottom) using data from TCGA. **Supplemental Figure S4.** Analysis of the prognostic significance of *RALA* expression in the METABRIC cohort. Patient expression data was stratified at the indicated preselection cutoffs and the resulting Kaplan Meier curves were compared by log-rank. Corresponding Log-rank *P*-values are shown in each panel. *RALA* was prognostic of DSS at all preselection points. **Supplemental Figure S5.** Analysis of the prognostic significance of *RALA* expression in the TNBC population of the METABRIC cohort over a 5 year follow-up period. TNBC patient samples were dichotomized into high (upper 10^th^ percentile) and low (lower 90^th^ percentile). The resulting Kaplan Meier curves were compared statistically by log-rank. **Supplemental Figure S6.** Analysis of the prognostic significance of *RALB* expression in the METABRIC cohort. Patient expression data was stratified at the indicated preselection cutoffs and the resulting Kaplan Meier curves were compared by log-rank. Corresponding log-rank *P*-values are shown in each panel. *RALB* was prognostic of DSS when the population was stratified along the median *RALB* expression (lower left panel), or when the upper two-tertiles were compared to the remaining lowest tertile (bottom, middle panel). A trend toward significance remained when the upper three-quartiles were compared to the lowest quartile (bottom right panel). **Supplemental Figure S7.** Analysis of the prognostic significance of expression of RAL effectors in the METABRIC cohort. The METABRIC cohort was stratified along median expression for *EXOC2* (**A**), *EXOC8* (**B**), or *RALBP1* (**C**) and the resulting Kaplan Meier curves were compared by log-rank. **Supplemental Figure S8. (A and B)** Analysis of the prognostic significance of expression of *RALGAPA1* and *RALGAPA2* in the METABRIC cohort. The METABRIC cohort was stratified along median expression for (A) *RALGAPA1* or (B) *RALGAPA2* and the resulting Kaplan Meier curves were compared by log-rank. **(C and D)** *RALGAPA1* (C) or *RALGAPA2* (D) expression in samples of normal breast, TNBC, or other BC subtypes were compared statistically. **(E)** The METABRIC cohort was stratified along median expression for *RALGAPB* and the resulting Kaplan Meier curve was compared by log-rank. **(F)** *RALGAPB* expression in the METABRIC dataset was compared statistically across normal breast, TNBC, or other BC subtypes. *; *P* < 0.05 **Supplemental Figure S9. (A - D)** Analysis of the prognostic significance of expression of (A) *RALGDS*, (B) *RGL1*, (C) *RGL2*, and (D) *RGL3* in the METABRIC cohort. The METABRIC cohort was stratified along median expression for each respective gene and the resulting Kaplan Meier curves were compared by log-rank. **(E)** *RGL3* expression in the METABRIC dataset was compared statically across normal breast, TNBC, or other BC subtypes. *; *P* < 0.05 **Supplemental Figure S10. (A and B)** Quantification of MDA-MB-468 cell viability upon treatment with varying doses of BQU57 under (A) normal adherent growth conditions for 72 h or (B) under low adhesion (GILA) conditions for 5 days. **(C and D)** Mouse body weight was monitored over the course of BQU57 treatment in mice bearing (C) MDA-MB-231 or (D) TNBC PDX tumors.

**Additional file 2.**

## Data Availability

Not applicable.

## Abbreviations

BC: Breast cancer
DAB: 3,3'-Diaminobenzidine
DSS: Disease-specific survival
ER: Estrogen receptor
GAP: GTPase-activating protein
GEF: Guanine nucleotide exchange factors
H&E: Hematoxylin and eosin
HER2: Human epidermal growth factor receptor 2
IACUC: Institutional Animal Care and Use Committee
IHC: Immunohistochemistry
MTT: 3-(4,5-Dimethylthiazole-2-yl)-2,4-diphenyltetrazolium bromide
NSG: NOD-scid-gamma
OS: Overall survival
OSUCCC: Ohio State University Comprehensive Cancer Center
PDX: Patient-derived xenograft
PR: Progesterone receptor
TCGA: The Cancer Genome Atlas Program
TMA: Tissue microarray
TNBC: Triple-negative breast cancer
ULAR: University Laboratory Animal Resources
