# Supplementary material for: The small G-protein RalA promotes progression and metastasis of triple-negative breast cancer
Source: Breast Cancer Res. 2021 Jun 12;23:65. doi: 10.1186/s13058-021-01438-3 (PMC8196523; doi:10.1186/s13058-021-01438-3)
Supplement: Supplementary file 1 — Additional file 1: Supplemental Figure S1. (A) Representative low magnification images of lungs harvested from mice bearing MDA-MB-231 Ctrl, RALA-KO, or RALB-KO tumors. Scale bar = 2000μm. (B and C) Representative images and quantification of lung nodules resulting from MDA-MB-231 shCtrl (n = 4) and shRALA (n = 4) orthotopic mammary tumors (B) or shCtrl (n = 10) and shRALB (n = 12) tumors (C). Tumor-bearing mice were sacrificed when the respective shCtrl group met ERC. (D) Representative images and H-score quantification of Ki67 immunostaining in lung tumor nodules from mice bearing MDA-MB-231 shCtrl or shRALA tumors. Error bars represent SEM; *, P < 0.05. Supplemental Figure S2. (A) Western blots demonstrating RALA and RALB expression in MDA-MB-468 siRNA control (siCtrl), siRALA, and siRALB cells. (B) Representative images and quantification of MDA-MB-231 shCtrl, shRALA and shRALB cell migration after 6 h (scale bar = 200μm). (C) Representative images and quantification of MDA-MB-231 shCtrl, shRALA and shRALB cell invasion through matrigel coated transwell inserts after 24 h (scale bar = 200μm). (D) Western blots demonstrating RALA and RALB expression for MDA-MB-231 Ctrl, RALA-KO, and RALB-KO cells ± siCtrl or siRALA/B transient knockdown. (E) Quantification of viability for MDA-MB-231 shCtrl, shRALA, and shRALB cells. (F) Quantification of viability for MVT1 shCtrl and shRALA cells over 72 h. (G) Quantification of BrdU incorporation for MDA-MB-231 shCtrl, shRALA, and shRALB cells over 72 h. (H) Quantification of BrdU incorporation for MVT1 shCtrl and siRALA cells over 72 h. (I) Quantification of Annexin V positivity for MDA-MB-231 shCtrl, shRALA, and shRALB cells. (J) Quantification of Annexin V positivity for MVT1 shCtrl and siRALA cells. (K) Quantification of growth in low adhesion (GILA) conditions for MDA-MB-231 shCtrl, shRALA and shRALB cells for 5 days. (L) Quantification of growth in low adhesion (GILA) conditions for MVT1 shCtrl and shRALA cells for 5 days [file 13058_2021_1438_MOESM1_ESM.pdf]

S1

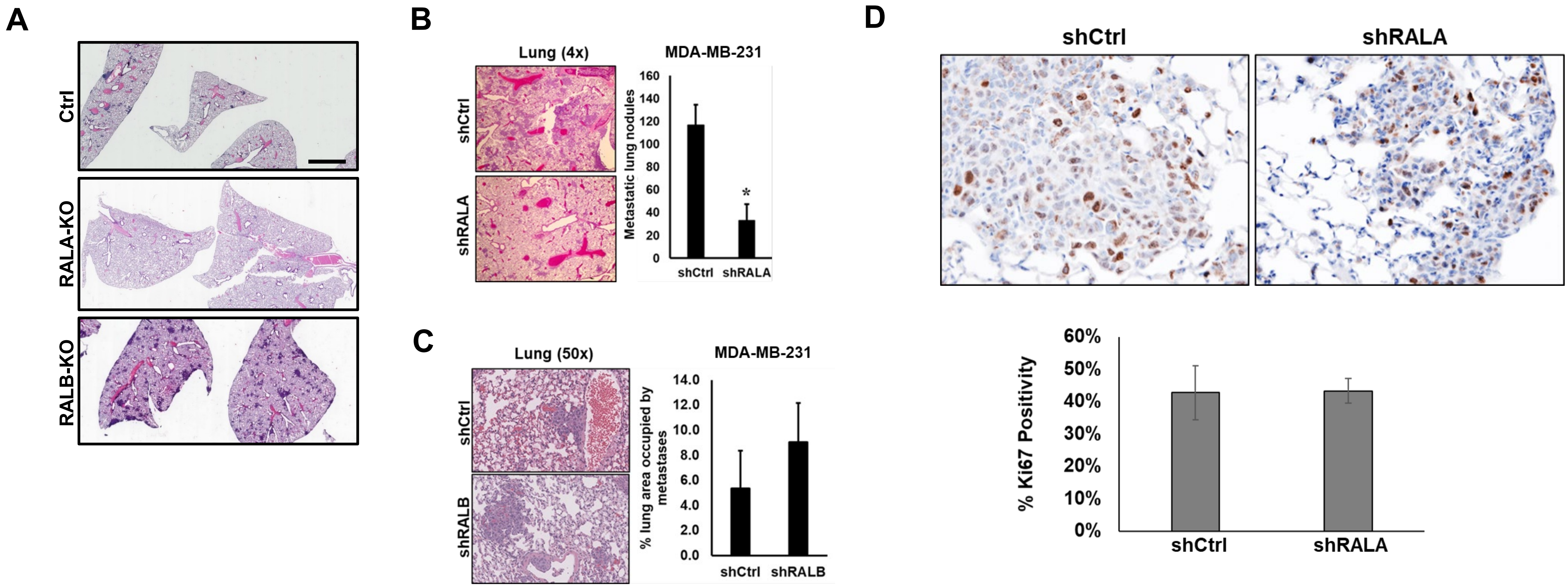
A
Ctrl
RALA-KO
RALB-KO
B
Lung (4x)
MDA-MB-231
shCtrl
shRALA
Metastatic lung nodules
160
140
120
100
80
60
40
20
0
*
shCtrl
shRALA
C
Lung (50x)
MDA-MB-231
shCtrl
shRALB
% lung area occupied by metastases
14.0
12.0
10.0
8.0
6.0
4.0
2.0
0.0
shCtrl
shRALB
D
shCtrl
shRALA
% Ki67 Positivity
60%
50%
40%
30%
20%
10%
0%
shCtrl
shRALA

S2

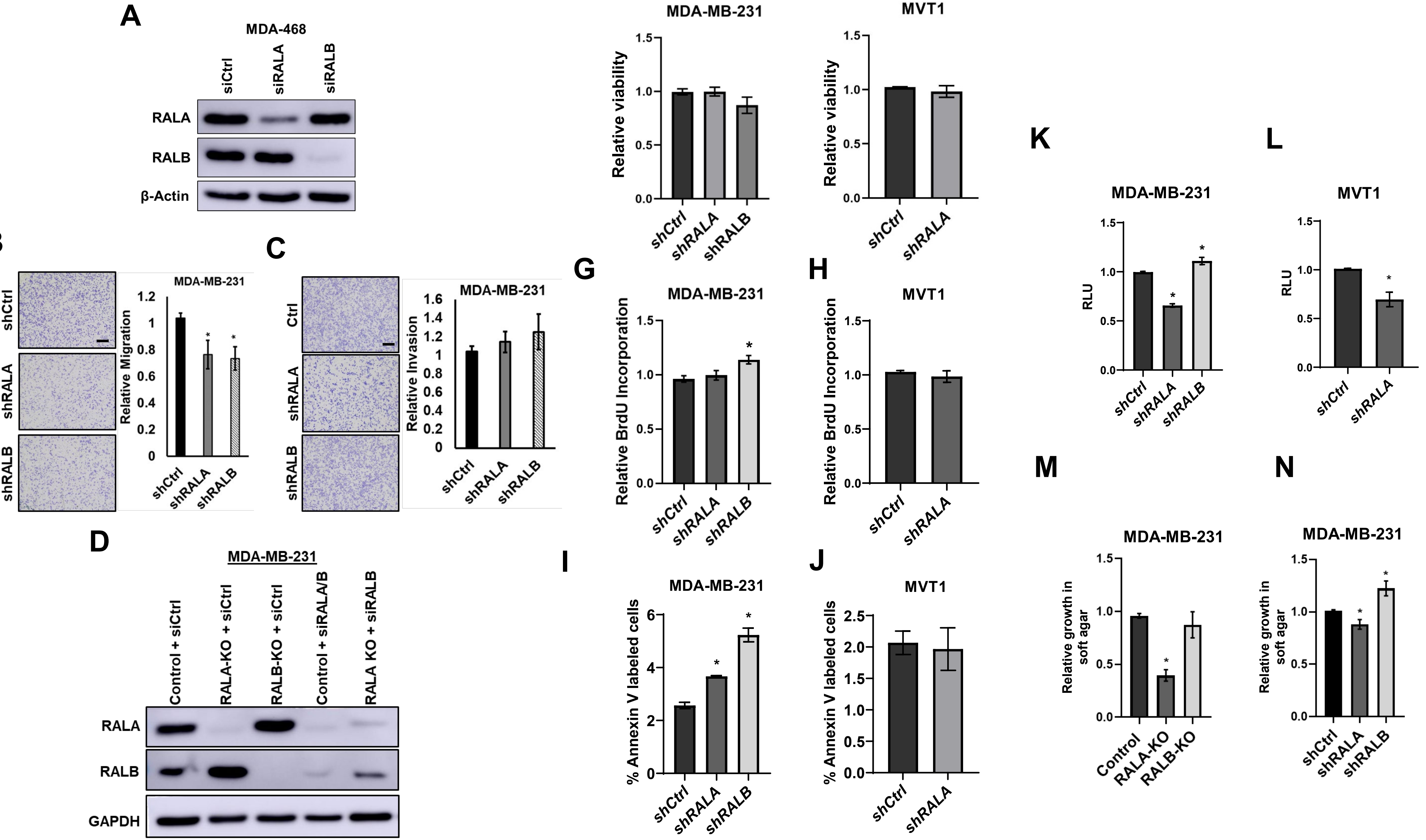

A
MDA-468
siCtrl
siRALA
siRALB
RALA
RALB
β-Actin
B
shCtrl
shRALA
shRALB
MDA-MB-231
Relative Migration
C
Ctrl
shRALA
shRALB
MDA-MB-231
Relative Invasion
D
MDA-MB-231
Control + siCtrl
RALA-KO + siCtrl
RALB-KO + siCtrl
Control + siRALA/B
RALA KO + siRALB
RALA
RALB
GAPDH
E
MDA-MB-231
Relative viability
F
MVT1
Relative viability
G
MDA-MB-231
Relative BrdU Incorporation
H
MVT1
Relative BrdU Incorporation
I
MDA-MB-231
% Annexin V labeled cells
J
MVT1
% Annexin V labeled cells
K
MDA-MB-231
RLU
L
MVT1
RLU
M
MDA-MB-231
Relative growth in soft agar
Control
RALA-KO
RALB-KO
N
MDA-MB-231
Relative growth in soft agar

S3

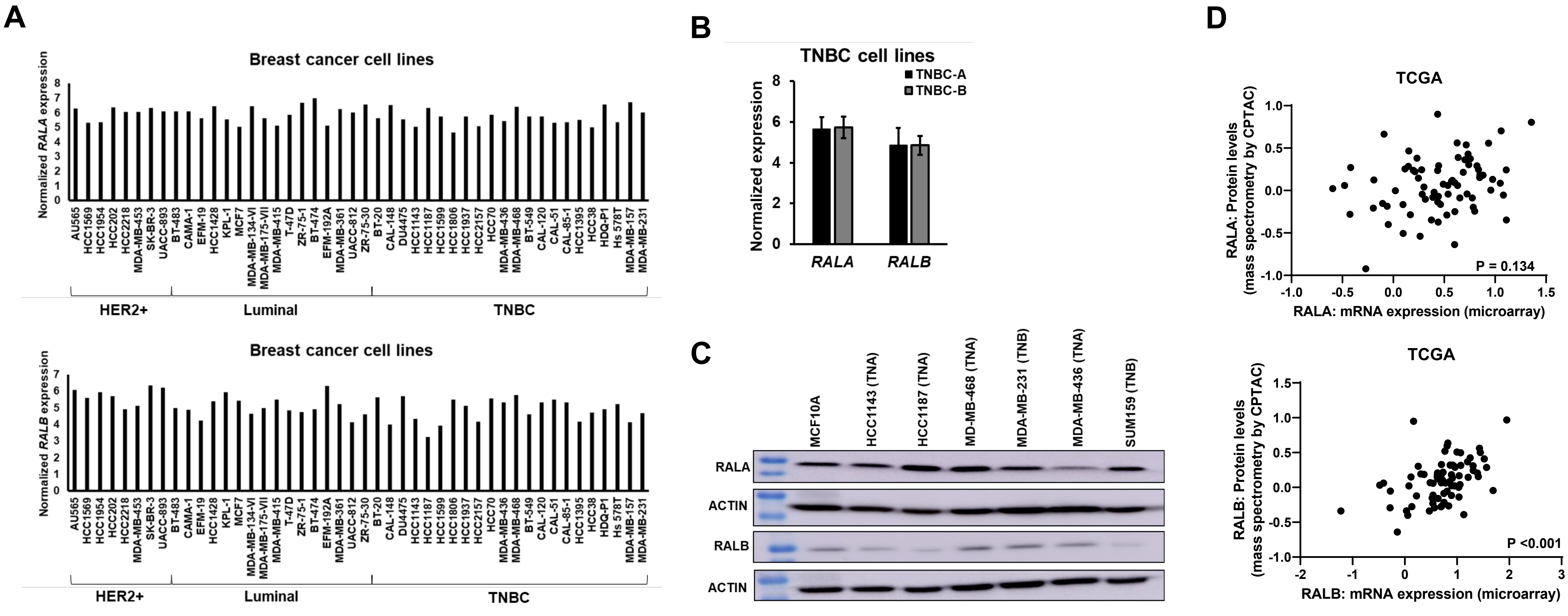
A
Breast cancer cell lines
Normalized RALA expression
Normalized RALB expression
HER2+
Luminal
TNBC
AU565
HCC1569
HCC1954
HCC202
HCC2218
MDA-MB-453
SK-BR-3
UACC-893
BT-483
CAMA-1
EFM-19
HCC1428
KPL-1
MCF7
MDA-MB-134-VI
MDA-MB-175-VII
MDA-MB-415
T-47D
ZR-75-1
BT-474
EFM-192A
MDA-MB-361
UACC-812
ZR-75-30
BT-20
CAL-148
DU4475
HCC1143
HCC1187
HCC1599
HCC1806
HCC1937
HCC2157
HCC70
MDA-MB-436
MDA-MB-468
BT-549
CAL-120
CAL-51
CAL-85-1
HCC1395
HCC38
HDQ-P1
Hs 578T
MDA-MB-157
MDA-MB-231
B
TNBC cell lines
Normalized expression
TNBC-A
TNBC-B
RALA
RALB
C
MCF10A
HCC1143 (TNA)
HCC1187 (TNA)
MD-MB-468 (TNA)
MDA-MB-231 (TNB)
MDA-MB-436 (TNA)
SUM159 (TNB)
RALA
ACTIN
RALB
ACTIN
D
TCGA
RALA: Protein levels (mass spectrometry by CPTAC)
RALA: mRNA expression (microarray)
P = 0.134
RALB: Protein levels (mass spectrometry by CPTAC)
RALB: mRNA expression (microarray)
P <0.001

S4

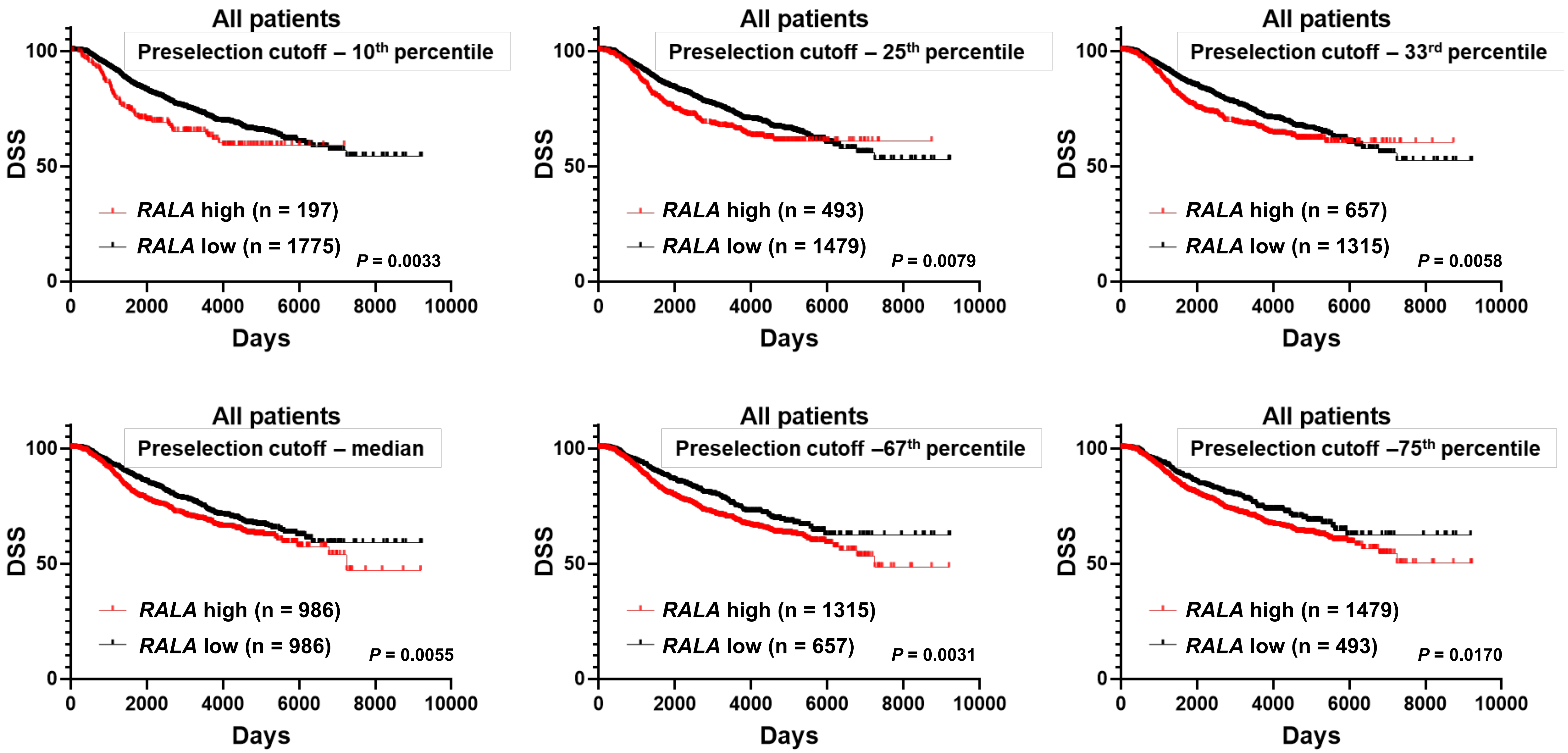
All patients
Preselection cutoff – 10th percentile
DSS
100
50
0
RALA high (n = 197)
RALA low (n = 1775)
P = 0.0033
0
2000
4000
6000
8000
10000
Days
All patients
Preselection cutoff – 25th percentile
DSS
RALA high (n = 493)
RALA low (n = 1479)
P = 0.0079
Days
All patients
Preselection cutoff – 33rd percentile
DSS
RALA high (n = 657)
RALA low (n = 1315)
P = 0.0058
Days
All patients
Preselection cutoff – median
DSS
RALA high (n = 986)
RALA low (n = 986)
P = 0.0055
Days
All patients
Preselection cutoff –67th percentile
DSS
RALA high (n = 1315)
RALA low (n = 657)
P = 0.0031
Days
All patients
Preselection cutoff –75th percentile
DSS
RALA high (n = 1479)
RALA low (n = 493)
P = 0.0170
Days

S5

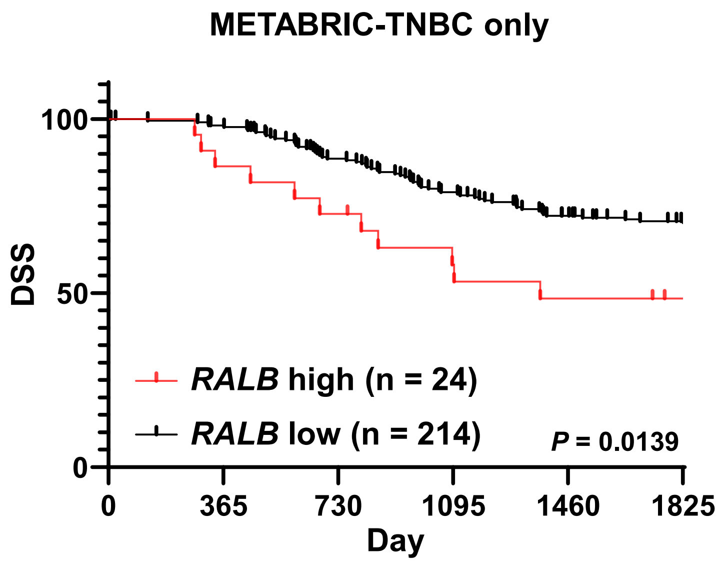
METABRIC-TNBC only
100
50
0
DSS
0
365
730
1095
1460
1825
Day
RALB high (n = 24)
RALB low (n = 214)
P = 0.0139

S6

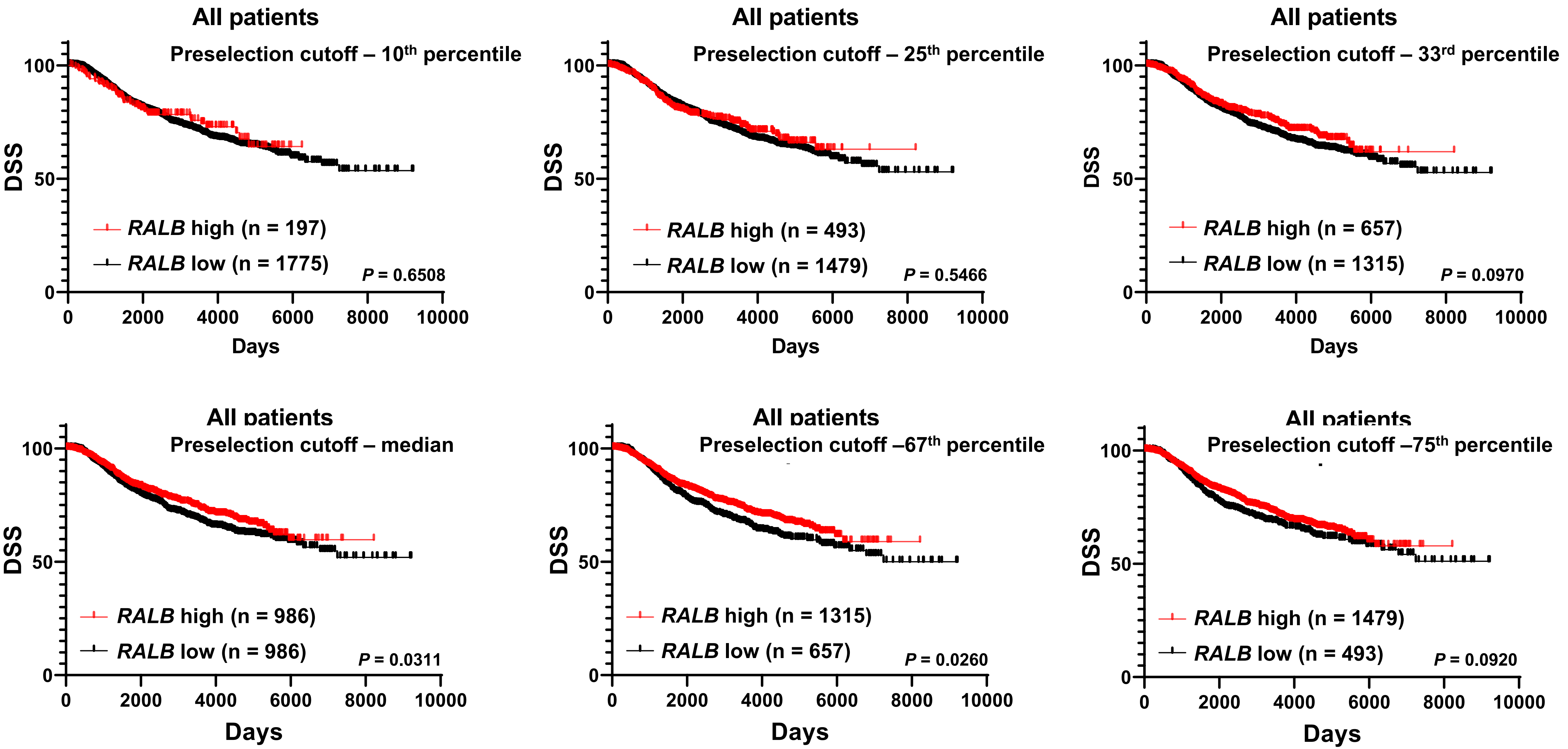
All patients
Preselection cutoff – 10th percentile
DSS
Days
RALB high (n = 197)
RALB low (n = 1775)
P = 0.6508
All patients
Preselection cutoff – 25th percentile
RALB high (n = 493)
RALB low (n = 1479)
P = 0.5466
All patients
Preselection cutoff – 33rd percentile
RALB high (n = 657)
RALB low (n = 1315)
P = 0.0970
All patients
Preselection cutoff – median
RALB high (n = 986)
RALB low (n = 986)
P = 0.0311
All patients
Preselection cutoff –67th percentile
RALB high (n = 1315)
RALB low (n = 657)
P = 0.0260
All patients
Preselection cutoff –75th percentile
RALB high (n = 1479)
RALB low (n = 493)
P = 0.0920

S7

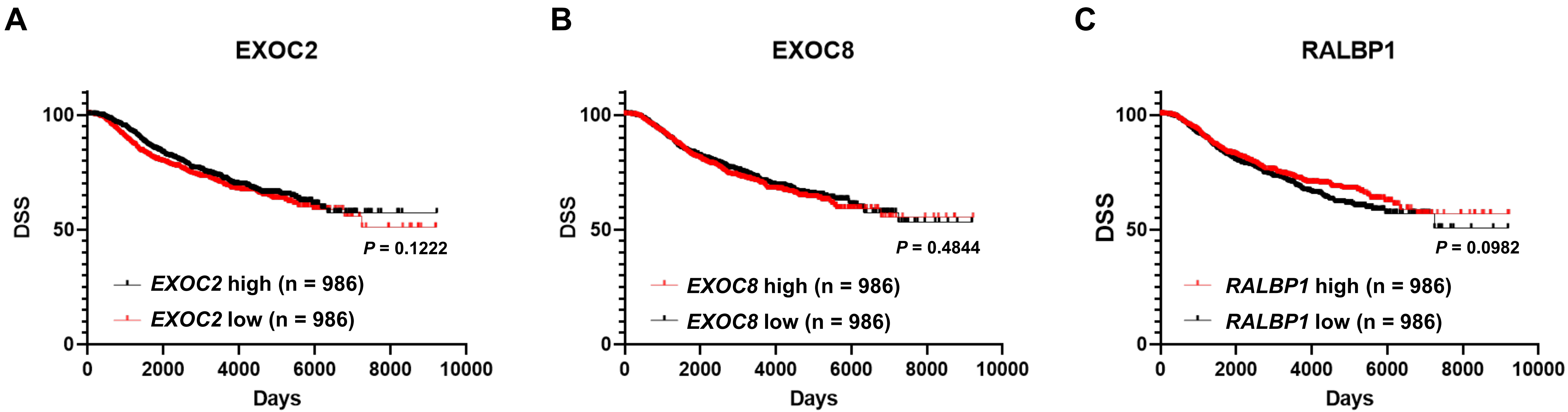
A
EXOC2
DSS
100
50
0
P = 0.1222
EXOC2 high (n = 986)
EXOC2 low (n = 986)
0
2000
4000
6000
8000
10000
Days
B
EXOC8
DSS
100
50
0
P = 0.4844
EXOC8 high (n = 986)
EXOC8 low (n = 986)
0
2000
4000
6000
8000
10000
Days
C
RALBP1
DSS
100
50
0
P = 0.0982
RALBP1 high (n = 986)
RALBP1 low (n = 986)
0
2000
4000
6000
8000
10000
Days

S8

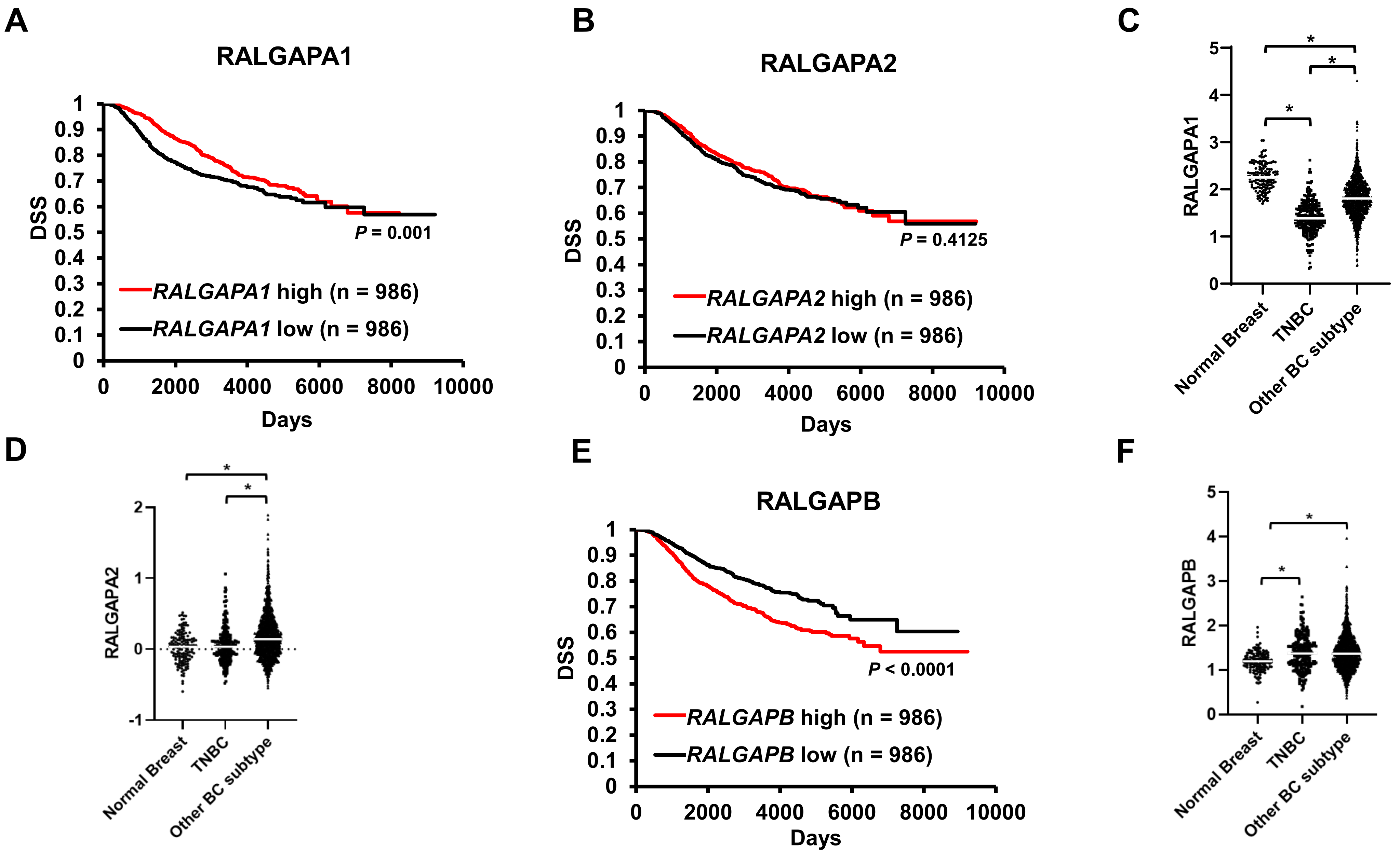
A
RALGAPA1
DSS
Days
P = 0.001
RALGAPA1 high (n = 986)
RALGAPA1 low (n = 986)
B
RALGAPA2
DSS
Days
P = 0.4125
RALGAPA2 high (n = 986)
RALGAPA2 low (n = 986)
C
RALGAPA1
Normal Breast
TNBC
Other BC subtype
D
RALGAPA2
Normal Breast
TNBC
Other BC subtype
E
RALGAPB
DSS
Days
P < 0.0001
RALGAPB high (n = 986)
RALGAPB low (n = 986)
F
RALGAPB
Normal Breast
TNBC
Other BC subtype

S9

Analysis of RALGEFs as a prognosticators of DSS in the METABRIC cohort

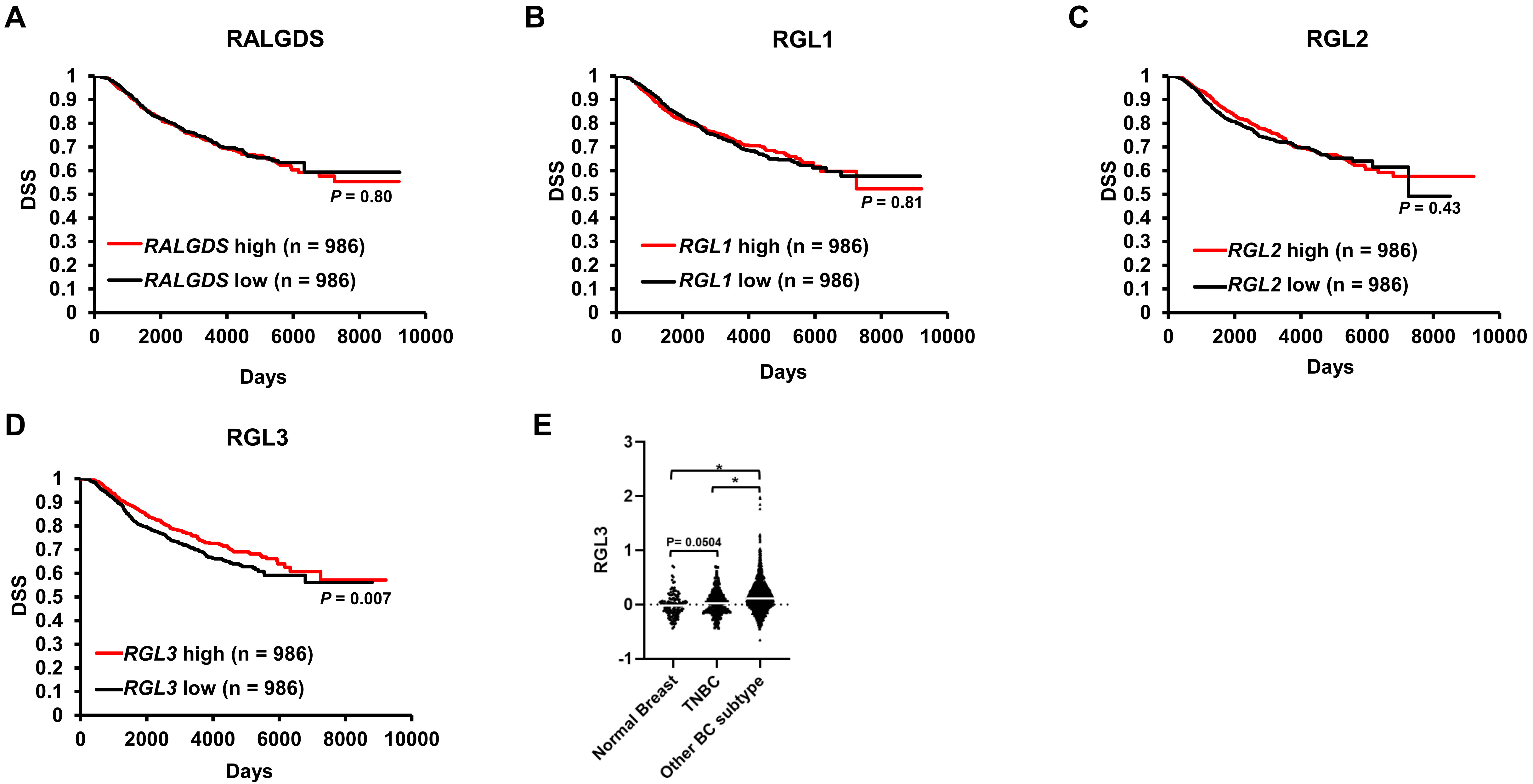

S10

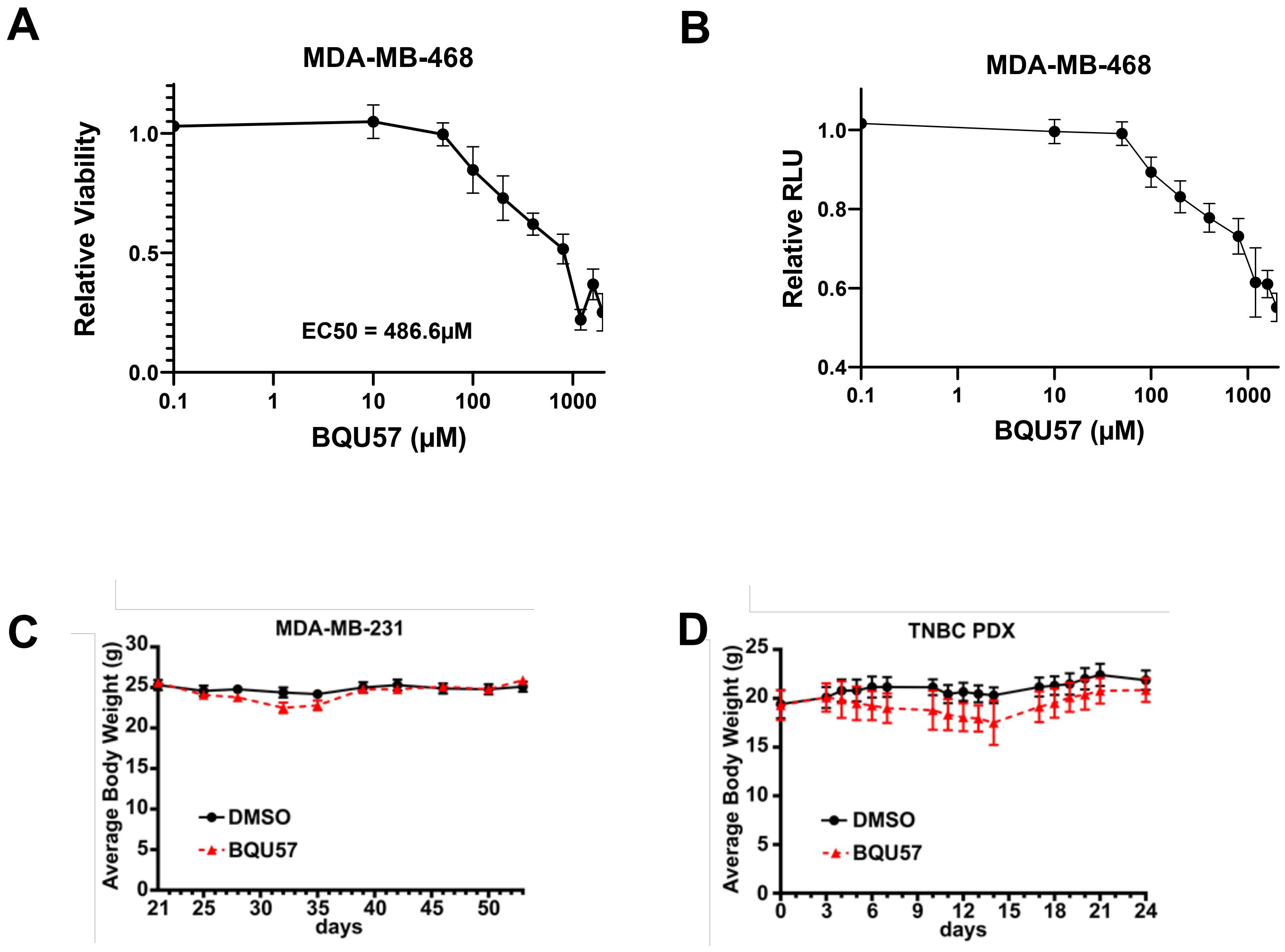
A
MDA-MB-468
Relative Viability
1.0
0.5
0.0
EC50 = 486.6μM
0.1
1
10
100
1000
BQU57 (μM)
B
MDA-MB-468
Relative RLU
1.0
0.8
0.6
0.4
0.1
1
10
100
1000
BQU57 (μM)
C
MDA-MB-231
Average Body Weight (g)
30
25
20
15
10
5
0
DMSO
BQU57
21
25
30
35
40
45
50
days
D
TNBC PDX
Average Body Weight (g)
25
20
15
10
5
0
DMSO
BQU57
0
3
6
9
12
15
18
21
24
days
